# RNA pull-down confocal nanoscanning (RP-CONA) detects quercetin as pri-miR-7/HuR interaction inhibitor that decreases α-synuclein levels

**DOI:** 10.1093/nar/gkab484

**Published:** 2021-06-09

**Authors:** Siran Zhu, Nila Roy Choudhury, Saul Rooney, Nhan T Pham, Joanna Koszela, David Kelly, Christos Spanos, Juri Rappsilber, Manfred Auer, Gracjan Michlewski

**Affiliations:** Infection Medicine, University of Edinburgh, The Chancellor’s Building, Edinburgh EH16 4SB, UK; Dioscuri Centre for RNA-Protein Interactions in Human Health and Disease, International Institute of Molecular and Cell Biology in Warsaw, Warsaw 02-109, Poland; Infection Medicine, University of Edinburgh, The Chancellor’s Building, Edinburgh EH16 4SB, UK; Infection Medicine, University of Edinburgh, The Chancellor’s Building, Edinburgh EH16 4SB, UK; School of Biological Sciences, IQB3, University of Edinburgh, Edinburgh EH9 9FF, UK; School of Biological Sciences, IQB3, University of Edinburgh, Edinburgh EH9 9FF, UK; The Wellcome Centre for Cell Biology, University of Edinburgh, Michael Swann Building, Max Born Crescent, Edinburgh EH9 3BF, UK; The Wellcome Centre for Cell Biology, University of Edinburgh, Michael Swann Building, Max Born Crescent, Edinburgh EH9 3BF, UK; The Wellcome Centre for Cell Biology, University of Edinburgh, Michael Swann Building, Max Born Crescent, Edinburgh EH9 3BF, UK; Department of Biotechnology, Technische Universität Berlin, Berlin 13355, Germany; School of Biological Sciences, IQB3, University of Edinburgh, Edinburgh EH9 9FF, UK; Dioscuri Centre for RNA-Protein Interactions in Human Health and Disease, International Institute of Molecular and Cell Biology in Warsaw, Warsaw 02-109, Poland; Infection Medicine, University of Edinburgh, The Chancellor’s Building, Edinburgh EH16 4SB, UK; Zhejiang University-University of Edinburgh Institute, Zhejiang University School of Medicine, Zhejiang University, Haining, Zhejiang 314400, P.R. China

## Abstract

RNA–protein interactions are central to all gene expression processes and contribute to a variety of human diseases. Therapeutic approaches targeting RNA–protein interactions have shown promising effects on some diseases that are previously regarded as ‘incurable’. Here, we developed a fluorescent on-bead screening platform, RNA Pull-Down COnfocal NAnoscanning (RP-CONA), to identify RNA–protein interaction modulators in eukaryotic cell extracts. Using RP-CONA, we identified small molecules that disrupt the interaction between HuR, an inhibitor of brain-enriched miR-7 biogenesis, and the conserved terminal loop of pri-miR-7–1. Importantly, miR-7′s primary target is an mRNA of α-synuclein, which contributes to the aetiology of Parkinson’s disease. Our method identified a natural product quercetin as a molecule able to upregulate cellular miR-7 levels and downregulate the expression of α-synuclein. This opens up new therapeutic avenues towards treatment of Parkinson’s disease as well as provides a novel methodology to search for modulators of RNA–protein interaction.

## INTRODUCTION

RNA–protein interactions coordinate the whole of RNA metabolism, including transcription, RNA processing, modification, translation and turnover (1). Dysregulated RNA metabolism can result in serious diseases including cancer (2) and neuropathological conditions (3). In recent years, therapies targeting RNA–protein interactions have shown promising results and some of them are already in clinical trials (4–6). Among many types of RNAs, short non-coding microRNAs (miRNAs, miRs) that regulate gene expression by imperfect base-pairing to mRNA hold great potential both as biomarkers (7) and therapeutics (8) in multiple human pathologies. The hairpin containing transcripts, known as primary miRNAs (pri-miRNAs), are processed by the Microprocessor complex to become stem-loop precursor miRNAs (pre-miRNAs) (9–13). The intermediates are further cut by an RNase III enzyme DICER, generating miRNA duplexes (14,15). Only the final product, the single-stranded mature miRNAs together with the Argonaute proteins are functional regulators of gene expression (16).

Parkinson’s disease (PD) is an incurable neurodegenerative disease that affects all ages but is most prevalent in the elderly population, with over 1% of those over the age of 60 suffering from this disease (17). One of the main causes behind PD is overproduction and aggregation of a protein called α-synuclein (α-Syn), expressed from the SNCA gene in the brain cells of affected individuals (18–21). There is a large body of evidence that decreasing the levels of α-Syn should be beneficial for PD patients, and several clinical trials are now focusing on α-Syn clearance with small molecules, antibodies or vaccines (22). Notably, miR-7 has been shown to target α-Syn production (23) and is significantly downregulated in the substantia nigra pars compacta (SNpc) of PD patients (24). MiR-7 also targets other genes implicated in PD, including RelA, Sir2 or Nlrp3 (25). For these reasons, approaches for miR-7 replacement therapies have been put forward (26).

The biogenesis of miRNAs is tightly controlled through RNA–protein interactions (27–31). Our group has identified that an RNA-binding protein (RBP) HuR specifically binds to the Conserved Terminal Loop (CTL) of primary miR-7–1 (pri-miR-7–1). By recruiting another RBP MSI2, HuR increases the rigidity of the pri-miR-7–1 stem loop, therefore, blocking Microprocessor cleavage and preventing production of mature miR-7 (32). HuR-mediated inhibition of miR-7 biogenesis was independently reported by Lebedeva *et al.* (33). We have also found that the monounsaturated fatty acid-oleic acid (OA), previously found to bind to MSI2 (34), can dissociate the HuR/MSI2 complex from pri-miR-7–1 and facilitate the biogenesis of miR-7 (35). However, OA is not effective at micromolar concentrations, has poor bioavailability when tested in cells and is toxic in high concentrations (36). Thus, it is necessary to identify potent pri-miR-7–1/HuR inhibitors that will elevate miR-7 levels and lead to downregulation of its targets, including α-Syn, which could provide alternative solutions to PD therapy.

Here, we developed a fluorescent on-bead screening assay based on RNA Pull-Down Confocal Nanoscanning (RP-CONA) to identify small molecules that modulate the strength of RNA–protein interactions. Our method uses an ultra-sensitive RNA–protein pull-down assay in cell extracts from human cultured cells (37) detecting RNA–protein complex modulators by confocal microscopy (38–41). We have demonstrated that the method works for various RNA–protein complexes and screening platforms. By employing RP-CONA, we identified quercetin, a natural flavonoid and known inhibitor of HuR/TNF-α mRNA interaction (42), as the most potent pri-miR-7–1/HuR interaction inhibitor. Quercetin induces miR-7 level and inhibits α-Syn expression in an HuR-dependent fashion. In summary, in this study we introduce RP-CONA as a new assay technique for the identification of small molecule modulators of RNA–protein interactions. We identified novel inhibitors of HuR/RNA complexes and have discovered a validated hit compound towards attenuation of α-Syn levels in PD.

## MATERIALS AND METHODS

### Cell culture

HeLa and HEK293T cells were maintained in DMEM (Dulbecco’s Modified Eagle’s Medium, Gibco) containing 10% foetal bovine serum (Gibco). Transfection was performed using Lipofectamine^TM^ 2000 Transfection Reagent (Invitrogen) following the manufacturer’s instructions.

### Chemicals

The in-house 54-compound library was kindly provided by Professor Neil Carragher (The University of Edinburgh), including FDA-approved drugs and natural products of well-established anti-cancer mechanisms. The concentrations of library compounds were varied from 0.1 to 10 mM according to their optimal effects in previous cell studies in the Carragher’s laboratory. Ro 08–2750 was purchased from R&D Systems. Oleic acid, quercetin, luteolin, genistein, dihydrotanshinone I (DHTS), CMLD-2, cetylpyridinium chloride (CPC) and gossypol were purchased from Sigma-Aldrich. All nine reagents were dissolved in DMSO to prepare 20 mM stock solutions.

### Plasmid construction

Human genomic DNA was isolated from HeLa cells using a GenElute™ Mammalian Genomic DNA Miniprep Kit (Sigma-Aldrich). Target genes were PCR amplified from genomic DNA using Phusion^®^ High-Fidelity DNA Polymerase (NEB) and propagated following the instructions of CloneJET PCR Cloning Kit (Thermo). The HuR open reading frame was inserted downstream from the mCherry gene between XhoI and EcoRI in a pJW99 plasmid. DNA segments encoding miRNA stem loop sequences were cloned into a pCG plasmid (32) between the XbaI and BamHI cleavage sites. The 3′-UTR of human α-Syn mRNA gene was inserted into a psiCHECK-2 plasmid between the XhoI and NotI sites downstream the Renilla luciferase (Rluc) gene. Sequence confirmation was carried out in Edinburgh Genomics or GENEWIZ.

### Mutagenesis PCR

Mutants of psiCHECK-2-α-Syn-3′UTR were generated using mutagenesis PCR, where the nucleotides in the third position of the putative miRNA binding sites were mutated. Primers used for mutagenesis are as follows: miR-7_s1-F: accatcagcagtgattgaagt; miR-7_s1-R: agacttcgagatacactgtaaa; miR-7_s2-F: attaatgatactgtctaagaataatg; miR-7_s2-R: agacacctaaaaatcttataatatat; miR-7_s3-F: acccttaatatttatctgacggta; miR-7_s3-R: agaaacactttaaaggagaatttg. PCR reactions were run for 18 cycles using Phusion polymerase. The template plasmids were digested by DpnI (NEB) treatment at 37°C for 1.5 h, followed by T4 Polynucleotide Kinase (NEB) treatment at 37°C for 1 h. Blunt-end ligation was performed using T4 DNA Ligase (NEB) at 4°C overnight. The ligation products were transformed and propagated in *Escherichia coli*.

### Western blot analysis

Cultured cells were harvested and resuspended in Roeder D (200 mg/ml glycerol, 100 mM KCl, 0.2 mM EDTA, 100 mM Tris pH 8.0, 500 μM DTT and 200 μM PMSF). Cell lysis was carried out in a Bioruptor® Plus sonication device (Diagenode) for 10 min (low intensity settings, 30 s on/off). Sixty micrograms of proteins in cell lysates were separated on a NuPAGE™ 4–12% Bis-Tris Protein Gel (Invitrogen) and transferred onto a nitrocellulose membrane (GE) in a GENIE® blotter (Idea Scientific) at 12 V for 1 h. The membrane was blocked with 1:10 Western Blocking Reagent (Roche) in TBST (20 mM Tris pH 7.5, 137 mM NaCl and 0.1% (v/v) Tween 20). Proteins were detected with the following primary antibodies in TBST containing 1:20 Western Blocking Reagent, including rabbit polyclonal anti-HuR (Millipore), rabbit polyclonal DHX9 antibody (Proteintech), monoclonal anti-α-tubulin antibody (Sigma-Aldrich) and purified mouse anti-α-Synuclein (BD Biosciences). Following three washes in TBST, the membrane was incubated in the horseradish peroxidase (HRP) conjugated secondary anti-rabbit or anti-mouse IgG antibodies (Cell Signalling Technology) and developed with chemiluminescent substrate (Thermo #34580). Quantification of western blot bands were carried out in Image Studio Lite Ver 5.2.

### RNA quantification

Total RNA was isolated from cells or cell extracts following manufacturer’s instruction of TRI Reagent™ Solution (Invitrogen) or TRI Reagent^®^ LS (Sigma-Aldrich), respectively. To quantify miRNA levels, reverse transcription and quantitative real-time PCR (qRT-PCR) were performed with miScript II RT Kit and SYBR Green PCR Kit respectively (QIAGEN). To quantify α-Syn mRNA levels, the GoTaq® 1-Step RT-qPCR System (Promega) was used with the primers (F: 5′-gttgtggctgctgctgagaaa; R: 5′-tccctccttggttttggagcctac). The qRT-PCR reactions were performed in a Roche LightCycler^®^ 96 System.

### Dual luciferase assays

Luciferase reporter assays were performed according to the manufacturer’s instruction of Dual-Luciferase^®^ Reporter Assay System (Promega). 1.2 × 10^4^ of HeLa cells were plated in a well of 96-well plates. For each well, 30 ng of psiCHECK2-α-Syn-3′UTR were co-transfected with 16.7 ng of pCG-pri-miRNA or 50 ng pCDNA-HuR plasmids in HeLa cells. Cells were lysed 48 hours after transfection by Passive Lysis Buffer (Promega). The luminescence levels of firefly and Renilla luciferases were recorded by a PolarStar OPTIMA Multidetection Microplate Reader (BMG LABTECH). The mRNA levels of luciferases were determined by qRT-PCR with following primers (Renilla-F: ggaatgggtaagtccggcaa; Renilla-R: ccaagcggtgaggtacttgt; firefly-F: gaacagctctgggtctaccg; firefly-R: gggatgatctggttgccgaa).

### Generation of gene knockout cells

To knockout HuR in HEK293T and HeLa cells, a pair of guide RNAs (5′-cgaagucuguucagcagcau and 5′-cuugggucauguucugaggg) targeting the exon2 of human HuR gene were designed. The Alt-R® CRISPR-Cas9 crRNAs and tracRNA were synthesized by IDT. 100 μM of each crRNA was mixed with 100 μM of tracRNA in 100 μl duplex buffer (IDT) at 95°C for 5 min to form two crRNA–tracRNA duplexes. HEK293T or HeLa cells were seeded in a 24-well plate. 1.5 μl of each duplex were co-transfected with 1 μl of GeneArt™ CRISPR Nuclease mRNA (Invitrogen). The cells were diluted and aliquoted to 96-well plates to make <1 cell count per well and lysed by 30 μl of Passive Lysis Buffer (Promega) when confluent. 2 μl of the cell extracts were loaded onto nitrocellulose membranes and tested against HuR and DHX9 antibodies. Cells showing HuR negative and DHX9 positive were selected and confirmed using western blot analysis. The genomic DNA of the knockouts were extracted and fragments covering the expected HuR knockout sites were PCR amplified using the forward primer (5′-gccctggacagtacactcgcc) and reverse primer (5′-ccacatggccgaagactgca). After ligating into the cloning vector using the CloneJET PCR Cloning Kit (Thermo), the DNA fragments were sequenced, and the mutations were identified.

To knockout pri-miR-7–1 in HeLa cells, a pair of guide RNAs (5′-acauucaauacuaaucuugc and 5′-accaaucauuuguccuguag) was designed flanking the stem loop sequence of human pri-miR-7–1 gene. Transfection of the CRISPR-Cas9 system was carried out as described before. Crude DNA was extracted by dissolving cells in solution 1 (25 mM NaOH, 0.2 mM EDTA) at 98°C for 1 h and terminated by equal volume of solution 2 (40 mM Tris-HCl, pH 5.5). PCR was performed with primers flanking the targeted region (F: 5′-ctgcagaacaggtcagtttaagtt, R: 5′-tgcagaacacctatgaagcaga). The PCR products were visualized on an agarose gel and cells generating band shifts were selected. The miR-7 levels were tested by qRT-PCR. Sequences were determined using PCR products amplified from purified genomic DNAs of putative pri-miR-7–1 knockouts.

### RP-CONA

Ni-NTA agarose beads (Qiagen, 30250) were sieved using 100 μm cell strainers (Corning, 431752) and 120 μm pore size filters (Millipore, NY2H04700) to obtain a uniform bead size population. The sieved beads were washed with binding buffer (0.3 M NaCl, 20 mM HEPES pH 7.5, 0.01% Triton X-100) and resuspended to 10% slurry. For each reaction, 150 pmol of 6× His-streptavidin (ProteoGenix) was mixed with 5 μl of sieved beads (10%) in a total volume of 20 μl binding buffer at 4°C, 1000 rpm for 20 min. The biotinylated FITC-pri-miR-7–1-CTL (5′-FITC-uguuguuuuuagauaacuaaaucgacaacaaa-Biotin-3′) were synthesized by IDT. 40 pmol of RNA was mixed with 5 μl of the streptavidin beads (10%) in a total volume of 20 μl PBS containing 0.01% Triton X-100 at 4°C, 1000 rpm for 20 min.

HuR KO-HEK293T cells were plated in a p150 dish and transfected with 40 μg of pJW99-HuR plasmids. The cell lysates were obtained 24 h after transfection by sonication. The concentration of mCherry-HuR in the lysates was quantified following the instruction of an mCherry quantification kit (BioVision). The lysates containing 300 nM of mCherry-HuR were diluted in 20 μl of no glycerol-Roeder D (100 mM KCl, 0.2 mM EDTA, 100 mM Tris pH 8.0, 0.5 mM DTT and 0.2 mM PMSF) and mixed with 30 μl of pulldown solution (1.5 mM MgCl_2_, 25 mM creatine-phosphate, 0.5 mM ATP, 0.25 μl Ribolock RNase Inhibitor (Invitrogen)) before loaded to a well of a black 384-well plate (SWISSCI or Greiner). 0.5 μl of compounds (dissolved in DMSO at 100 times of the final concentration) were added to the cell lysates and mixed vigorously at room temperature, 1500 rpm for 20 min.

5 μl of FITC-pri-miR-7-CTL-beads (10%) were added to the treated lysates and mixed at room temperature, 1500 rpm for 2 h. Images were acquired in an Opera® High Content Screening System (PerkinElmer) or ImageXpress Micro Confocal (Molecular Devices) at 30 μm above well bottom, 20 × magnification with air lenses. On the Opera HCS, three channels were detected: brightfield (690 nm diode at 70% power, 690/70 detection filter, 160 ms exposure time), FITC (488 nm laser at 250 μW, 520/35 detection filter, 120 ms exposure time) and mCherry (561 nm laser at 1250 μW, 600/40 detection filter, 240 ms exposure time). In ImageXpress, two channels were detected: FITC (FITC filter set: excitation 475/34, emission 536/40, exposure 25 ms) and mCherry (Texas Red filter set: excitation 560/32, emission 624/40, exposure 85 ms). FITC and mCherry rings were detected and the ring intensities were quantified in ImageJ, using a custom plugin (DOI:10.5281/ZENODO.4302193). *Z*’ factor was calculated using the equation *Z*’ = 1 – 3(SD _positive control_ + SD _negative control_)/ |Mean _positive control_ – Mean _negative control_| (43).

Other RP-CONA assays were conducted in the same manner. All the RNAs were synthesized by IDT, including FITC-pri-miR-7–1/30a-TL-bio (5′-FITC-uguuguucugugaagccacagaugggacaacaaa-Biotin-3′); Cy5-pre-let-7a-1-CTL-bio (5′-cy5-uuagggucacacccaccacugggagau-Biotin-3′); FITC-TNFα-ARE-bio (5′-FITC-auuauuuauuauuuauuuauuauuua-Biotin-3′); and FITC-αSyn-ARE-bio (5′-FITC-cuaaauccucacuauuuuuuuguugcug-Biotin-3′). Lin28-GFP was expressed using a pEGFP-N1 vector (44). Cy5 intensity was detected using Cy5 filter set (excitation 631/28, emission 692/40, exposure 2 ms) and GFP using FITC filter set (exposure 100 ms).

### RNA pull-down assay

RNA pull-down assay was carried out to detect binding of RNA-binding proteins, from whole cell extracts, to RNA immobilized on the beads. 500 pmol of pri-miR-7–1-CTL (5′-uguuguuuuuagauaacuaaaucgacaacaaa) was treated with 100 mM NaAc and 5 mM sodium (meta)periodate in 200 μl of water and rotated for 1 h at room temperature in the dark. The RNA was precipitated by adding 600 μl of 100% ethanol and 15 μl of 3 M NaAc in dry ice for 20 min, followed by centrifugation at 13 000 rpm, 4°C for 10 min. The RNA pellet was washed with 70% ethanol and resuspended in 500 μl of 100 mM NaAc pH 5. 200 μl of adipic acid dihydrazide-agarose (Sigma-Aldrich) was washed with 100 mM NaAc and mixed with 500 μl of the periodate oxidized pri-miR-7–1-CTL overnight at 4°C in the dark. The pri-miR-7–1-CTL-beads were washed by 2M KCl, Buffer G (20 mM Tris-HCl pH 7.5, 137 mM NaCl, 1 mM EDTA, 1% Triton X-100, 10% glycerol, 1.5 mM MgCl_2_, 1 mM DTT and 200 μM PMSF) and Roeder D, respectively.

250 μl of HeLa cell lysates containing 1 mg of total protein were pre-incubated with 100 μM of test compounds and 400 μl of pulldown solution at 37°C, 700 rpm for 20 min. The pri-miR-7–1-CTL-beads were incubated with the treated cell lysates, at 37°C, 1200 rpm for 30 min. After washing with Buffer G, the beads were mixed with 6 μl of NuPAGE™ Sample Reducing Agent, 15 μl of LDS Sample Buffer and 39 μl of water. Proteins captured by RNA were denatured at 70 °C, 1000 rpm for 10 min. 30 μl of the supernatant was loaded onto an SDS-PAGE gel and western blot was performed to detect the level of proteins.

### RNA immunoprecipitation (RIP) assay

HuR-bound pri-miR-7–1 and α-Syn mRNA were immunoprecipitated following a method developed from a previously published protocol (45). In brief, 1.4 × 10^6^ HuR-KO HeLa cells were plated in p100 dishes, treated with DMSO or 20 μM quercetin, and transfected with 500 ng of pCDNA-HuR. RNA/proteins were cross-linked using 1% formaldehyde and incubated at room temperature for 10 min with rocking. Crosslinking reactions were stopped by the addition of 0.25 M glycine (pH 7.0) followed by incubation at room temperature for 5 min. The cells were resuspended in 500 μl of RIPA buffer (50 mM Tris–Cl, pH 7.5, 1% Nonidet P-40 (NP-40), 0.5% sodium deoxycholate, 0.05% SDS, 1 mM EDTA, 150 mM NaCl), sonicated and centrifuged to obtain cell lysates as described above. The protein levels were determined using the Pierce® BCA Protein Assay Kit (Thermo). 50 μl of Dynabeads™ Protein A for Immunoprecipitation (Invitrogen) were coupled with 3 μl of HuR antibody in BWB (0.02% Tween-20 in PBS) for 30 min at room temperature with rocking, then washed with RIPA buffer. The extracts containing 100 μg of protein were diluted with 500 μl of RIPA buffer, mixed with the antibody–coated beads at room temperature for 30 min. The beads were collected at 6000 *g* and the supernatant was kept for RNA extraction as loading control. The beads were washed five times with high-stringency RIPA buffer (50 mM Tris–Cl, pH 7.5, 1% NP-40, 1% sodium deoxycholate, 0.1% SDS, 1 mM EDTA, 1 M NaCl, 4 M urea and 0.2 mM PMSF). The beads containing the immunoprecipitated samples were collected and resuspended in 100 μl of 50 mM Tris–Cl, pH 7.0, 5 mM EDTA, 10 mM dithiothreitol (DTT) and 1% SDS. The beads were then incubated at 70°C for 45 min to reverse the crosslinking. The RNA was extracted from these samples using TRI Reagent® LS according to the manufacturers protocol. qRT-PCR was performed to detect pri-miR-7–1 and α-Syn mRNA levels.

### mRNA stability assay

About 1.44 × 10^5^ WT or HuR KO HeLa cells were plated in 12-well plates and treated with DMSO or 20 μM of quercetin, together with 10 μg/ml actinomycin D (Sigma-Aldrich). The cells were collected at 0, 6, or 12 h after actinomycin D treatment. Total RNA was isolated using TRI Reagent™. α-Syn mRNA and 18S levels were quantified using qRT-PCR.

## RESULTS

### MiR-7 is a major inhibitor of α-Syn expression

**Figure 1.**
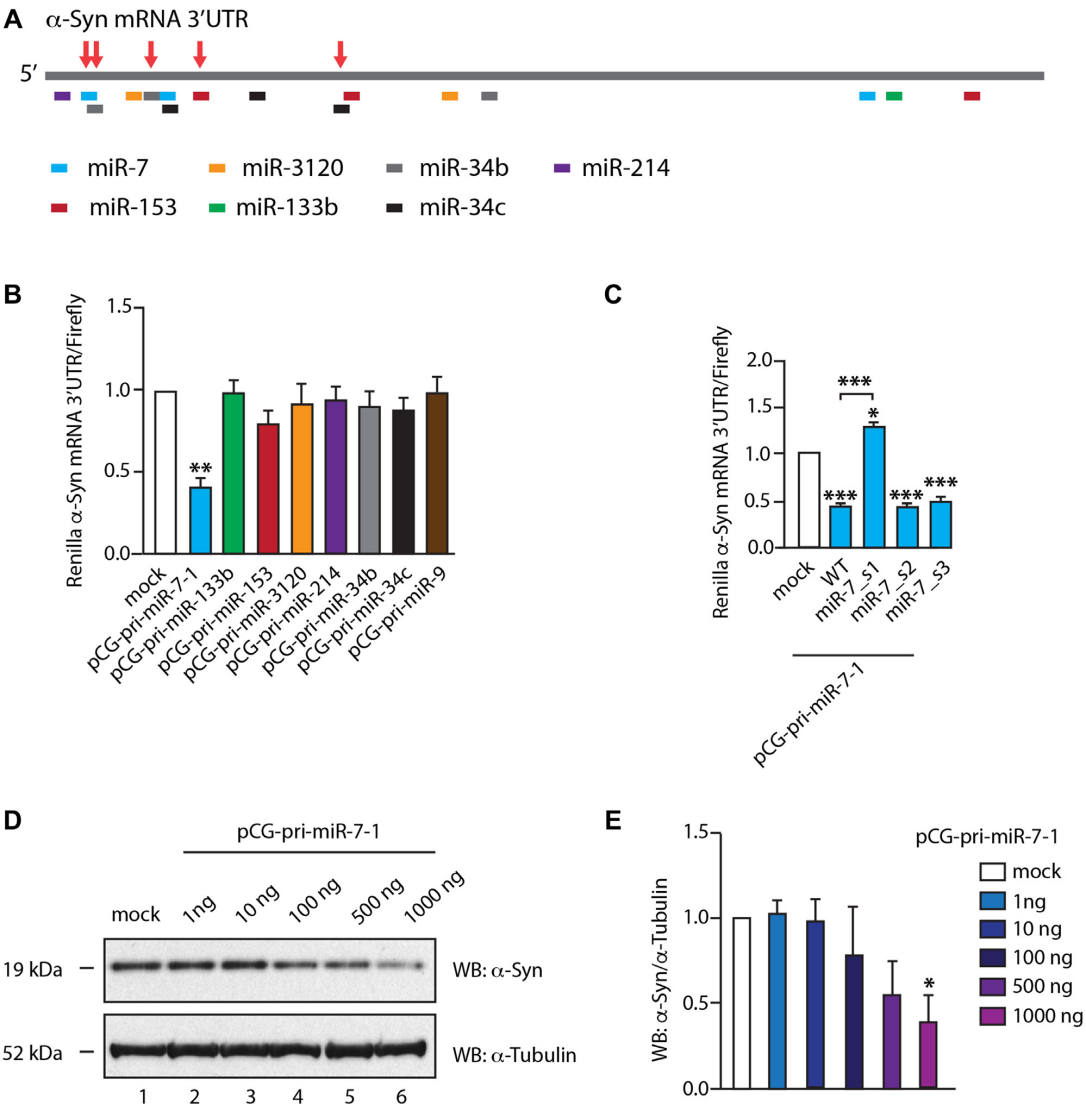
MiR-7 is a major suppressor of α-Syn expression. (**A**). Predicted binding sites of miRs on the 3′-UTR of α-Syn mRNA (∼2500 bp). The putative binding sites of different miRs are annotated at the approximate positions on the α-Syn mRNA 3′-UTR. These sites were predicted by TargetScan (47) or provided by miRTarBase (48). Sites highlighted by the red arrows were previously reported. (**B**). MiR-7 inhibited luminescence of a dual luciferase reporter bearing the gene of α-Syn mRNA 3′-UTR downstream the Renilla luciferase gene. Equal amount of each pCG-pri-miR plasmid was co-transfected with the luciferase reporter. PCG-pri-miR-9 was tested as a negative control. Luciferase levels were recorded 48 h after transfection. Mean Renilla/firefly values and SEM from six independent repeats are shown. (**C**). Single-nucleotide mutation of miR-7 binding site inactivated the inhibitive effects of miR-7. The nucleotide on the third position of miR-7 targeted seed regions on α-Syn mRNA 3′-UTR gene was mutated individually. The mutants were numbered according to the binding sites from 5′ to 3′ of α-Syn mRNA 3′-UTR. Luciferase levels were measured 48 h after the co-transfection of pCG-pri-miR-7–1 plasmids with reporters bearing wild-type or mutated α-Syn mRNA 3′-UTR gene. The luciferase levels were relative to co-transfection of pCG-pri-miR-9 with wild-type reporter. Mean Renilla/Firefly values and SEM from three independent repeats are shown. (**D** and**E**). α-Syn expression was inhibited by upregulated miR-7. 1: Mock HeLa cells without DNA transfected. 2–6: An increasing amount of pCG-pri-miR-7–1 was transfected into HeLa cells. The expression of α-Syn and α-tubulin were detected by western blot 48 h after transfection. Relative α-Syn/α-tubulin levels were normalized to mock. Mean values and SEM from three independent repeats are shown. Statistically significant differences compared to mock were interpreted by SPSS one-way ANOVA, with post hoc LSD test, *P< 0.05, **P< 0.01, ***P< 0.001.

MiR-7 and other miRs, such as miR-133b, miR-153, miR-34b or miR-34c, which also have the potential to target α-Syn, have been shown to be downregulated in PD, thus allowing α-Syn overproduction and accumulation (23,25,46–48). We collated all validated and predicted miRs targeting the 3′-UTR of α-Syn mRNA and cloned their pri-miR sequences into the pCG expression vector (Figure 1A). We used a dual luciferase reporter assay in HeLa cells with overexpression of individual miRs targeting α-Syn mRNA 3′-UTR coupled with Renilla luciferase mRNA. This assay showed that only miR-7 exhibited significant inhibition (>50%) of Renilla luciferase compared to other miRs, when equal amounts of pri-miR plasmids were transfected (Figure 1B). The upregulation of mature miR-7 was equal or less when compared with other miRs (Supplementary Figure S1A). Moreover, a single-nucleotide mutation in the previously identified miR-7 binding site (miR-7_s1) could desensitize α-Syn mRNA 3′-UTR to miR-7 overexpression, while other miR-7 binding sites (miR-7_s2 and miR-7_s3) did not present significant differences between the wild-type and mutated reporters (Figure 1C). Interestingly, mutations of miR-133b_s1 and miR-153_s1 sites exerted significant upregulation of expression from the α-Syn mRNA 3′-UTR Renilla luciferase construct, proving that miR-133b and miR-153 are involved in regulating α-Syn levels, albeit to lesser extent and with more complex regulatory networks (Supplementary Figure S1B).

Importantly, a gradient of miR-7 overexpression exerted gradual and significant reduction of the expression of endogenous α-Syn in HeLa cells (Figure 1D, E), which is consistent with a previous publication (23). HeLa cells were chosen because according to Human Protein Atlas these cells have high basal levels of α-Syn transcripts when compared with most other cultured cells (49). Other miRs, exemplified by miR-153 and miR-133b, exerted no significant inhibitive effects on endogenous α-Syn levels, in spite of their previously reported function as α-Syn inhibitors (Supplementary Figure S1C) (50,51). In summary, we conclude that miR-7 is the most potent α-Syn suppressor and uses a well-defined target site on the α-Syn mRNA 3′-UTR.

### RP-CONA: a novel, lysate-based scanning microscopy on-bead screening platform for small molecules that modulate RNA-protein complexes

Due to the supremacy of miR-7 in inhibiting the expression of α-Syn, it will be crucial to pursue therapeutic approaches for PD through the miR-7/α-Syn pathway. We hypothesize that as the biogenesis of pri-miR-7–1 is inhibited by HuR through an interaction with the Conserved Terminal Loop (CTL), small molecules that dissociate HuR from miR-7 CTL will facilitate the production of mature miR-7, which in turn will contribute to a reduction of α-Syn levels (32).

**Figure 2.**
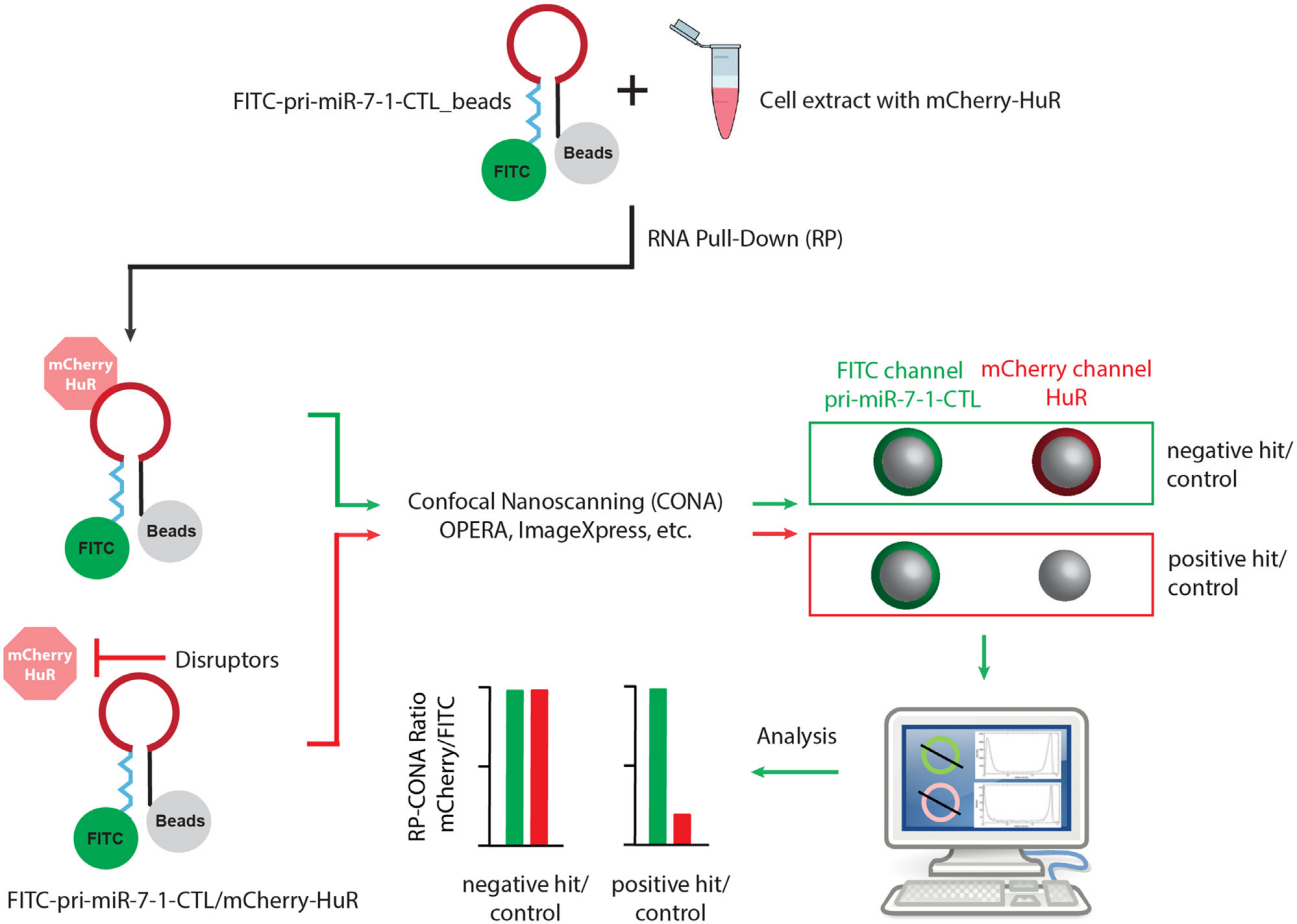
The working scheme of RP-CONA. RNA Pull-Down (RP): 5′-FITC-pri-miR-7–1-CTL-biotin-3′ is coupled to streptavidin coated agarose beads. Cell lysates are extracted from HuR KO HEK293T cells overexpressing mCherry-HuR and treated with small molecules. The RNA-coupled beads are incubated with cell lysates to pull-down mCherry-HuR. Confocal Nanoscanning (CONA): Beads are imaged using a confocal image scanning platform. On-bead FITC-pri-miR-7–1-CTL and mCherry-HuR are detected with high sensitivity as fluorescent rings/halos on the outer shell of bead in corresponding detection channels. Inhibitors are able to attenuate the mCherry fluorescence without affecting FITC signals. Analysis: The fluorescent rings are detected in ImageJ. Three measurements are taken across each ring and generate the fluorescent intensity profiles to obtain arbitrary intensity values.

To identify pri-miR-7/HuR inhibitors, we developed a screening platform that combines techniques of RNA Pull-Down (RP) from eukaryotic cell extracts, and on-bead scanning confocal microscopy (CONA), given the name RP-CONA (Figure 2). First, we attached 5′FITC and 3′biotin-tagged pri-miR-7–1-CTL to 6 × His-Streptavidin Ni-NTA agarose beads. Then we used HuR KO HEK293T cells, generated with CRISPR-Cas9 targeting, to overexpress mCherry-HuR (Supplementary Figure S2A–C). The HEK293T cells were chosen as they are known to support highly efficient recombinant protein production (52). Moreover, the HuR KO cells were used to avoid signal dilution from an untagged, endogenous HuR. Next, we performed RNA pull-down and imaged the fluorescently labelled RNA and mCherry-HuR on a confocal imaging system. Fluorescent rings/halos on the periphery of microbeads indicate binding events in separate detection channels. The amounts of bound RNA or protein were detected from fluorescence emission intensities of the rings/halos. Pri-miR-7–1-CTL/mCherryHuR inhibitors are expected to attenuate mCherry ring intensities without affecting FITC levels.

**Figure 3.**
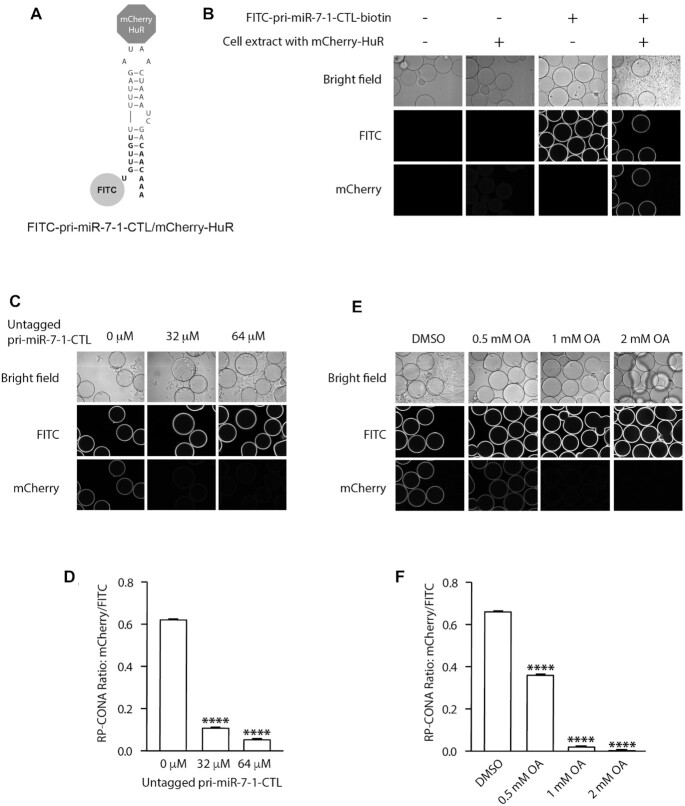
Optimization of RP-CONA method in the Opera HCS. (**A**) A diagram illustrating the interaction between FITC-pri-miR-7–1-CTL and mCherry-HuR. (**B**) Beads images of blank beads; blank beads incubated with cell lysates containing mCherry-HuR; FITC-pri-miR-7–1-CTL-beads incubated in lysates-free buffer; and FITC-pri-miR-7–1-CTL-beads after mCherry-HuR pulldown. (**C** and**D**) mCherry/FITC signals were reduced by untagged pri-miR-7–1-CTL. Cell lysates containing mCherry-HuR were treated with an increased concentration of untagged pri-miR-7–1-CTL before pull-down. (**E** and **F**) mCherry/FITC signals were reduced by a pri-miR-7–1/HuR inhibitor. Cell lysates containing mCherry-HuR were treated with DMSO or an increased concentration of OA before pull-down. All values were obtained from three technical repeats. Mean mCherry/FITC ring intensities and SD between triplicates are shown. Statistically significant differences compared to 0 μM untagged pri-miR-7–1 or DMSO were interpreted by SPSS independent sample *t*-test, ****P< 0.0001.

**Figure 4.**
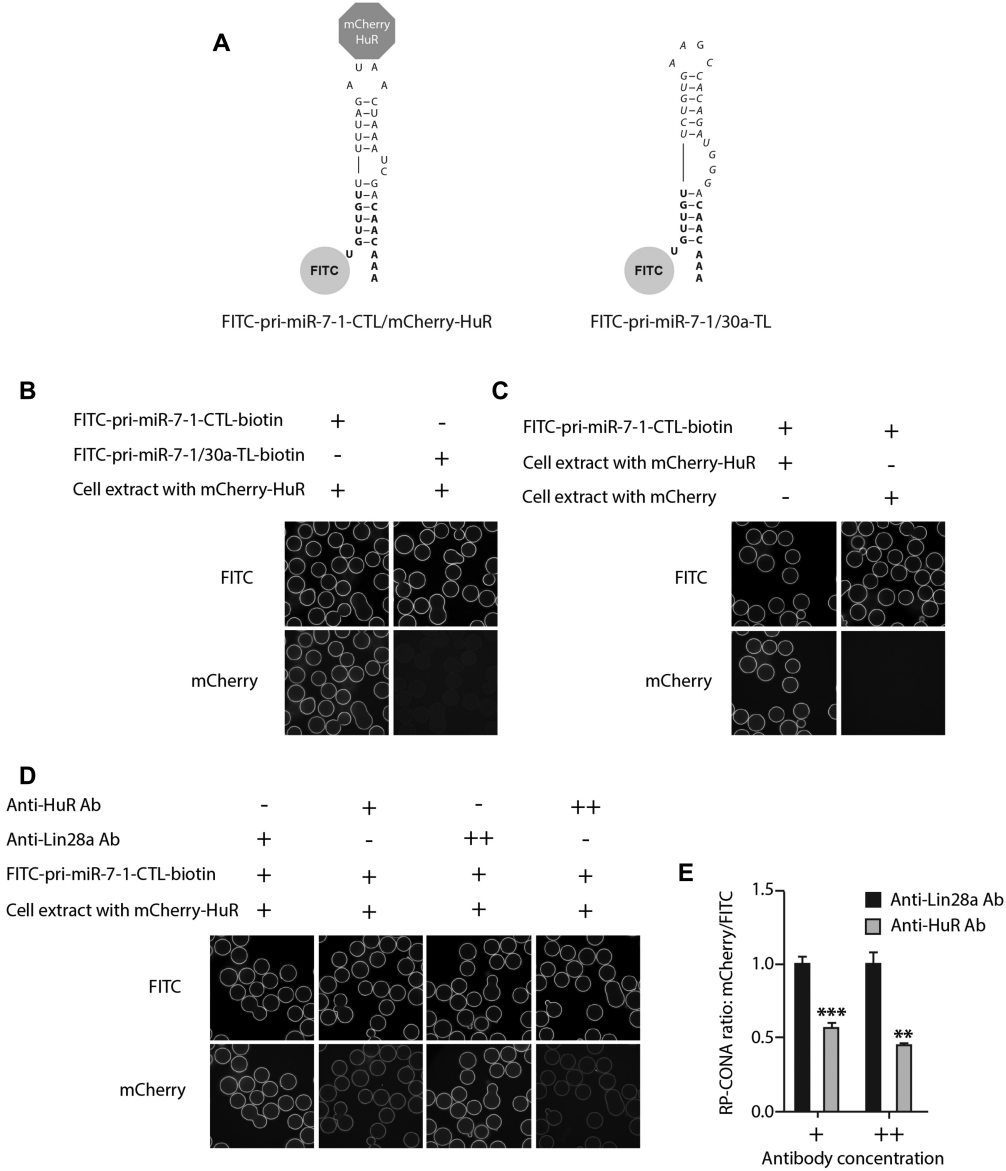
RP-CONA detects the specific interaction of pri-miR-7–1-CTL/HuR. (**A**) A diagram illustrating that mCherry-HuR interacts with FITC-pri-miR-7–1-CTL but not FITC-pri-miR-7–1/30a-TL. (**B**) mCherry-HuR pull-down by beads coupled with 40 pmol of FITC-pri-miR-7–1-CTL or FITC-pri-miR-7–1/30a-TL. (**C**) Pri-miR-7–1-CTL binds mCherry-HuR but not mCherry. FITC-pri-miR-7–1-CTL beads were incubated with cell extracts containing 300 nM mCherry-HuR or mCherry. (**D** and **E**) mCherry/FITC signals were reduced by anti-HuR antibody. Cell extracts containing 300 nM mCherry-HuR were pre-incubated with 2 μl (+) or 4 μl (++) of anti-Lin28a antibody (control) or anti-HuR antibody prior pulldown by FITC-pri-miR-7–1-CTL-beads. Mean mCherry/FITC ring intensities and SD between triplicates are shown. Statistically significant differences compared to anti-Lin28a antibody treated samples were interpreted by SPSS independent sample *t*-test, **P< 0.01, ***P<0.001.

We first tested the RP-CONA assay using the Opera HCS instrument. The preliminary results showed that RP-CONA generated FITC rings in the presence of the fluorescently tagged RNA, and mCherry rings only when mCherry-HuR was pulled down by pri-miR-7–1-CTL (Figure 3A, B). No overlapping signals were observed in different detection channels. The addition of an increasing concentration of untagged pri-miR-7–1-CTL competitively decreased mCherry signals (Figure 3C, D). Oleic acid, the known pri-miR-7–1/HuR complex inhibitor reduced mCherry ring intensities at high millimolar concentrations, as reported before (Figure 3E, F) (35). To show that RP-CONA can be run on various scanning instruments, we then switched to another confocal image platform, the ImageXpress, which gave similar quality of images compared to Opera HCS (Supplementary Figure S3A, B). Moreover, the relative mCherry/FITC value was proportional to the concentration of mCherry-HuR (Supplementary Figure S3C–E). Importantly, if we replaced the CTL of pri-miR-7–1 with the non-conserved Terminal Loop (TL) of pri-miR-30a that does not bind HuR (Figure 4A, B), or overexpressed mCherry instead of mCherry-HuR to make the cell extracts (Figure 4C), the mCherry rings were not observed. Moreover, the addition of polyclonal anti-HuR antibody to FITC-pri-miR-7–1-CTL/mCherry-HuR complex significantly decreased the mCherry ring intensities, compared with the control anti-Lin28a antibody (Figure 4D, E). This evidence proves that RP-CONA is a sensitive and specific method to probe pri-miR-7–1-CTL/HuR interactions.

**Figure 5.**
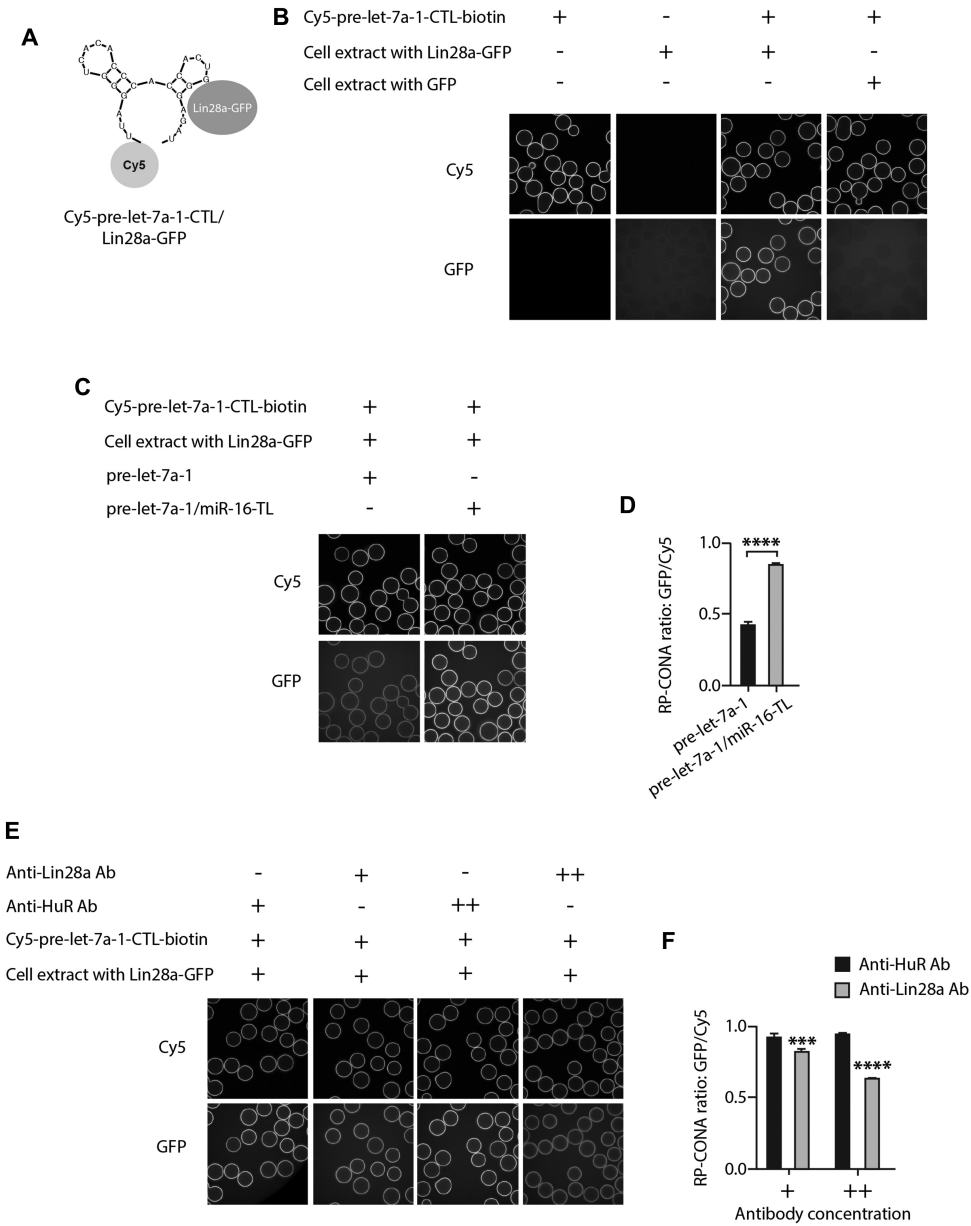
RP-CONA detects the specific interaction of pre-let-7a-1-CTL/Lin28a. (**A**) A diagram illustrating the interaction between Cy5-pre-let-7a-1-CTL and Lin28a-GFP. (**B**) Beads images of Cy5-pre-let7a-1-CTL-beads incubated in lysates-free buffer; blank beads incubated with cell lysates containing Lin28a-GFP; Cy5-pre-let7a-1-CTL-beads incubated with cell lysates containing Lin28a-GFP; and Cy5-pre-let-7a-1-CTL-beads incubated with cell lysates containing GFP. (**C** and**D**) GFP/Cy5 signals were reduced by unlabelled pre-let-7a-1. Cell extracts containing 300 nM of GFP-Lin28a were treated with 80 pmol of pre-let-7a-1 or pre-let-7a-1/miR-16-TL, prior to pull-down with Cy5-pre-let7a-1-CTL-beads. (**E** and**F**). GFP/Cy5 signals were reduced by anti-Lin28a antibody. Cell extracts containing 300 nM Lin28a-GFP were pre-incubated with 2 μl (+) or 4 μl (++) of anti-HuR antibody (control) or anti-Lin28a antibody prior pulldown by Cy5-pre-let-7a-1-CTL-beads. Mean GFP/Cy5 ring intensities and SD between triplicates are shown. Statistically significant differences compared to HuR antibody treated samples were interpreted by SPSS independent sample *t*-test, ***P< 0.001, ****P< 0.0001.

RP-CONA is also applicable for monitoring the interactions between other RNA-protein complexe, with different fluorophore pairs. To this end, we tested the well-established pre-let-7/Lin28a interaction (53–55) in RP-CONA, with the Cy5-tagged pre-let-7a-1-CTL and HEK293T cell extracts containing overexpressed Lin28a-GFP (Figure 5A, B; Supplementary Figure S4). The binding of Lin28a was significantly decreased by the competition of untagged pre-let-7a-1 but not the chimeric pre-let-7a-1/miR-16-TL (that does not bind Lin28a (44)), where the CTL of pre-let-7a-1 was replaced by the TL of miR-16 (Figure 5C, D). Notably, the Lin28a-GFP rings were significantly reduced by polyclonal anti-Lin28a antibody, compared to anti-HuR antibody (Figure 5E, F).

These results demonstrate that RP-CONA is a robust technique to screen for RNA–protein interaction modulators and that it could be used with various confocal image scanning platforms.

### Pri-miR-7/HuR inhibitors identified by RP-CONA screening of a focused library

**Figure 6.**
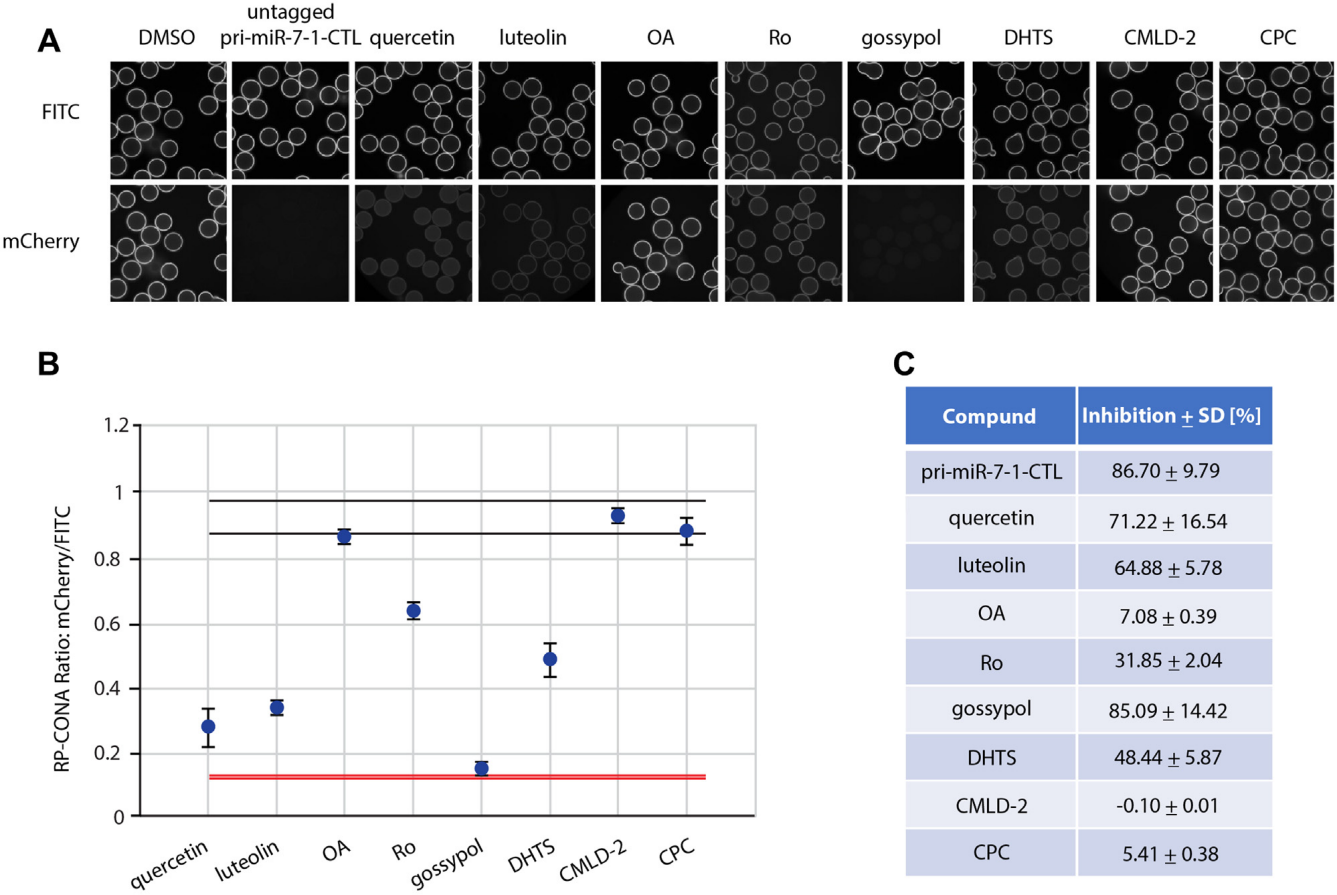
RP-CONA identified pri-miR-7–1/HuR inhibitors from a focused library. (**A**) Cell lysates containing mCherry-HuR were treated with DMSO, 50 μM of untagged pri-miR-7–1-CTL or 100 μM of compounds before pulldown. Beads images taken in ImageXpress. (**B**) Relative mCherry/FITC ring intensity mean and SD between the beads in each well after compound treatment are shown. DMSO (RP-CONA ratio: 0.932 ± 0.016, CV: 1.75%, *n* = 5) served as a negative control while 50 μM of untagged pri-miR-7–1-CTL (RP-CONA ratio: 0.124 ± 0.001, CV: 0.96%, *n* = 5) served as a positive control. *Z*’ = 0.93. Black lines: DMSO mean ± 3 × SD between five repeated wells. Red lines: untagged pri-miR-7–1-CTL mean ± 3 × SD between five repeated wells. At least 400 beads were included in each control analysis. (**C**). Inhibition of RP-CONA signals compared to DMSO. The percentage inhibition of compounds relative to DMSO mean are shown. The SD of relative inhibitions between the beads in each well after compound treatment are shown.

MSI2 and HuR inhibit miR-7 biogenesis and the level of DROSHA cleavage (32). A range of compounds have been recognized to inhibit HuR or MSI2 binding to their target mRNAs (6). By collecting the commercially available HuR/MSI2 inhibitors, we built up a focused library and tested them using RP-CONA at 100 μM concentration (Figure 6A). The *Z*’ factor of the screen is 0.93, and the coefficients of variation (CVs) of the negative control (DMSO) and positive control (untagged pri-miR-7–1-CTL) equal to 1.75% and 0.96%, respectively (Figure 6B). As reported previously (35), OA did not show significant disruptive effects on pri-miR-7–1/HuR complex at 100 μM. Importantly, we identified quercetin, luteolin and gossypol as pri-miR-7–1/HuR inhibitors, which generated >50% inhibition at 100 μM concentration according to the relative mCherry/FITC signals compared to the DMSO control (Figure 6C). In summary, RP-CONA identified new potential inhibitors of pri-miR-7–1-CTL/HuR complexes.

### A small-scale prototype RP-CONA screen of pri-miR-7-1/HuR inhibitors

**Figure 7.**
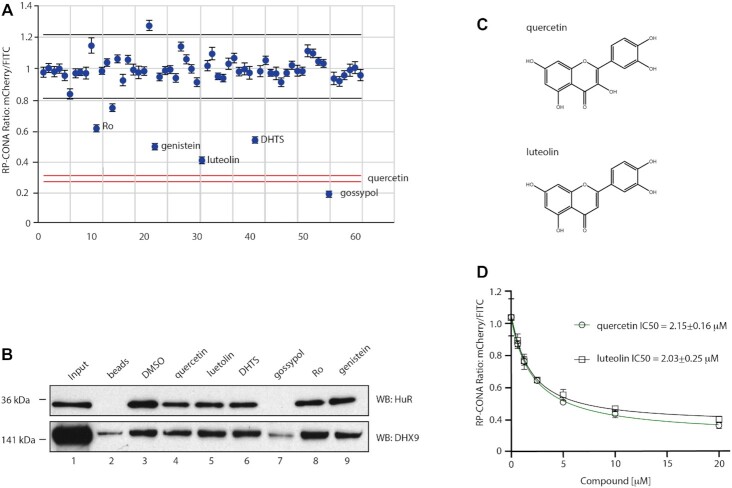
Identification and validation of pri-miR-7–1/HuR inhibitors from an enlarged library. (**A**) A small-scale prototype RP-CONA screen to test the disruptive effects of 61 compounds (54 from an in-house library varied from 1 to 100 μM, and seven from the previous identified HuR or MSI2 inhibitors at 100 μM) on pri-miR-7–1/HuR. Relative mCherry/FITC ring intensity mean and SD between the beads in each well after compound treatment are shown. DMSO (RP-CONA ratio: 1.009 ± 0.067, CV: 6.70%, *n* = 6) served as a negative control while 100 μM of quercetin (RP-CONA ratio: 0.288 ± 0.006, CV: 2.18%, *n* = 6) served as a positive control. *Z*’ = 0.69. Black lines: DMSO mean ± 3 × SD between six repeated wells. Red lines: quercetin mean ± 3 × SD between six repeated wells. At least 500 beads were included in each control analysis. Compounds generating >40% (six times CV of negative controls) inhibition are annotated. (**B**) Disruption of endogenous HuR pulled down by pri-miR-7–1-CTL. Pri-miR-7–1-CTL was covalently linked to agarose beads. HeLa extracts were treated with DMSO or 100 μM of each compound prior to pull-down. HuR and DHX9 in the pull-down components were detected by western blot. 1: Input. Unbound proteins in the lysates after pulldown. 2: Proteins pulled down by beads without RNA. 3–9: Proteins pulled down by pri-miR-7–1-CTL-beads after compound treatment. (**C**) Chemical structures of quercetin and luteolin. (**D**) Quercetin and luteolin disrupted pri-miR-7/HuR in a dose-dependent manner in RP-CONA. Increased concentrations of quercetin and luteolin were tested in RP-CONA. Relative mCherry/FITC ring intensity mean and SD between triplicated wells after compound treatment are shown. The RP-CONA signals were curve fitted by non-linear regression-four parameter [inhibitor] versus response, and IC50s were determined with the 4-parameter equation in GraFit v7.0.3 (Erithacus Software Limited) (40). The IC_50_s of quercetin and luteolin were 2.15 ± 0.16 μM and 2.03 ± 0.25 μM, respectively.

In order to analyse RP-CONA’s ability to scale up and to establish the sensitivity of the method, we performed a small-scale prototype screen using an in-house library containing 54 FDA-approved drugs or natural products, together with eight compounds from the previous focused screen (Figure 7A). Here, we applied quercetin as a positive control and DMSO as a negative control. The CVs of the negative and positive controls were 6.70% and 2.18%, respectively. Most compounds did not show significant stabilization or destabilization of the pri-miR-7–1-CTL/HuR complex. Additionally, we identified genistein as a new hit, albeit not as effective as quercetin or luteolin. To validate the active compounds identified in the primary screen, we tested them in a standard pull-down assay where pri-miR-7–1-CTL was covalently linked to the beads (56). Importantly, this assay, unlike RP-CONA, only gives semi-quantitative readout. The western blot analysis of the HuR pull-down confirmed the strong disruptive effects of quercetin and luteolin (Figure 7B). Similar effects from DHTS, Ro and genistein were also observed. Meanwhile, gossypol largely reduced pull-down of the RNA helicase DHX9, implying the effects of this compound are unspecific. Our most effective inhibitors quercetin and luteolin are close analogues (Figure 7C). We tested quercetin and luteolin in RP-CONA at a range of concentrations. The RP-CONA signals were reduced in a dose-dependent manner for both quercetin and luteolin, showing the half maximal inhibitory concentrations (IC_50_s) at 2.15 ± 0.16 μM and 2.03 ± 0.25 μM, respectively (Figure 7D). These observations indicate that RP-CONA primary hits were successfully validated using a classic biochemical method and further confirm quercetin and luteolin as the most promising inhibitors of the pri-miR-7–1/HuR complex.

### Quercetin inhibits cellular α-Syn and upregulates miR-7

**Figure 8.**
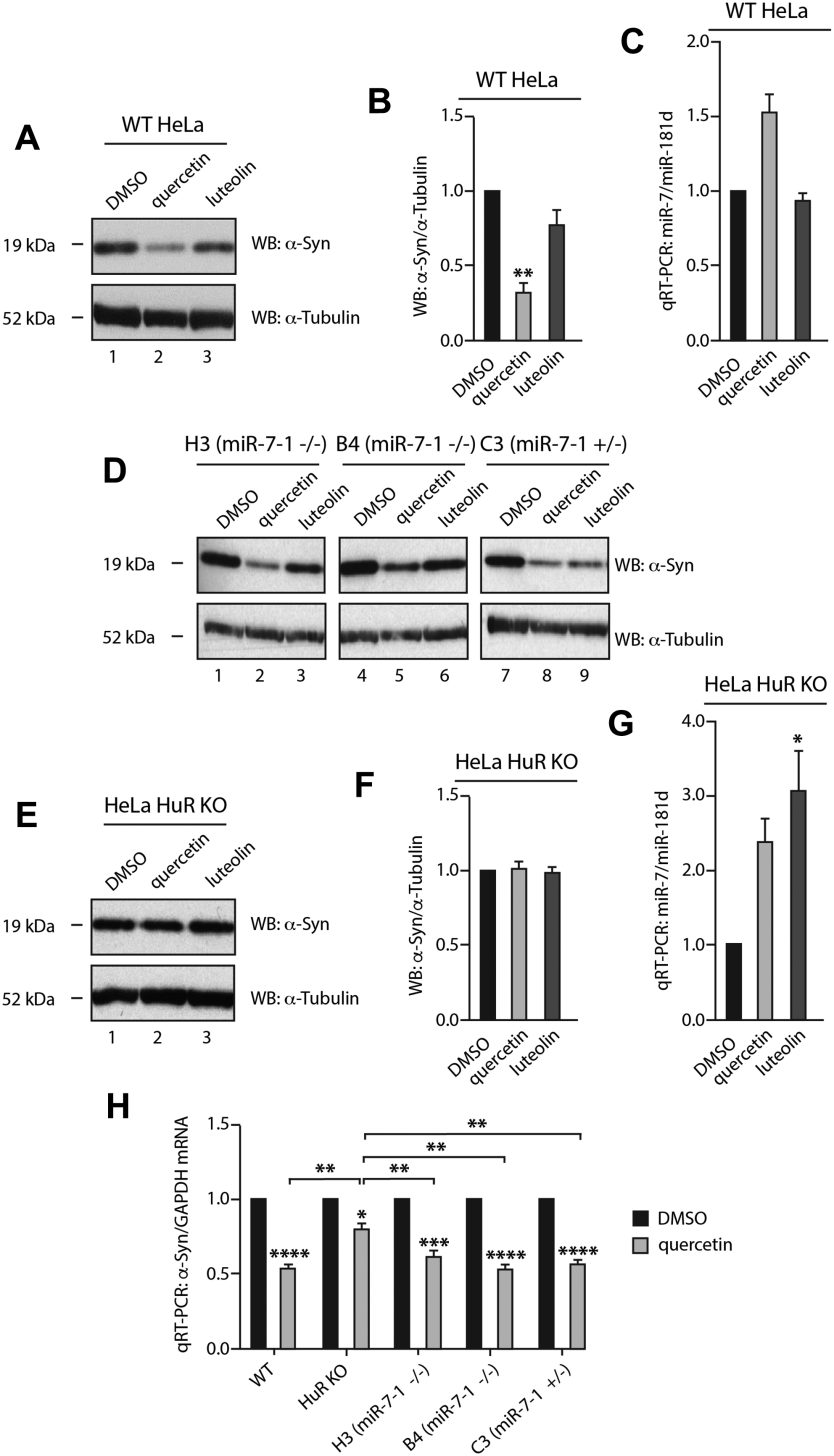
Quercetin inhibits cellular α-Syn and upregulates miR-7. (**A** and**B**) Quercetin significantly downregulates α-Syn expression in HeLa cells. HeLa cells were treated with 1: DMSO, 2: 20 μM of quercetin or 3: luteolin and harvested 48 h after treatment. Levels of α-Syn and α-tubulin were detected by western blot. Mean α-Syn/α-tubulin and SEM from three independent repeats are shown. (**C**) Quercetin upregulates cellular miR-7 level by 1.5-fold. HeLa cells were treated with DMSO, 20 μM of quercetin or luteolin and harvested 48 h after treatment. Mature miR-7 and miR-181d (housekeeping miR control) levels were determined by qRT-PCR. Mean miR-7/miR-181d and SEM from three independent repeats are shown. (**D**) Quercetin reduces α-Syn expression in a miR-7-independent pathway. HeLa miR-7–1 KO H3 (miR-7–1^−/−^), B4 (miR-7–1^−/−^) and C3 (miR-7–1^+/−^) were treated with 1, 4, 7: DMSO, 2, 5, 8: 20 μM of quercetin, or 3, 6, 9: luteolin for 48 h. Expression of α-Syn and α-tubulin were detected by western blot. (**E** and **F**) Quercetin reduces α-Syn expression in an HuR-dependent manner. HuR KO HeLa cells were treated with 1: DMSO, 2: 20 μM of quercetin or 3: luteolin and harvested 48 h after treatment. Levels of α-Syn and α-tubulin were detected by western blot. Mean α-Syn/α-tubulin and SEM from three independent repeats are shown. (**G**) Quercetin and luteolin upregulate cellular miR-7 level in HuR KO HeLa. HuR KO HeLa cells were treated with DMSO, 20 μM of quercetin or luteolin and harvested 48 h after treatment. Mature miR-7 and miR-181d levels were determined by qRT-PCR. Mean miR-7/miR-181d and SEM from three independent repeats are shown. (**H**) Quercetin downregulates α-Syn mRNA level in an HuR-dependent manner. Wild-type, HuR KO and miR-7–1 KO (H3, B4 and C3) HeLa were treated with DMSO or 20 μM of quercetin and harvested 48 h after treatment. α-Syn and GAPDH mRNA levels were determined by qRT-PCR. Mean α-Syn/GAPDH and SEM from three independent repeats are shown. Statistically significant differences compared to DMSO or between quercetin treated cells were interpreted by SPSS independent sample *t*-test, *P< 0.05, **P< 0.01, ***P< 0.001, ****P< 0.0001.

Subsequently, we tested quercetin and luteolin in HeLa cells at 20 μM concentration. Quercetin treated cells had a significantly reduced level of α-Syn protein, with a 1.5-fold upregulation of mature miR-7 level (Figure 8A–C). We found that quercetin induced pre-miR-7–1 but not pri-miR-7–1 levels >2-fold (Supplementary Figure S5A, B). The expression of DGCR8 and DICER were not significantly changed after quercetin treatment, albeit DROSHA levels were slightly upregulated (Supplementary Figure S5C). This suggests the amount of Microprocessor and DICER remained largely unaffected and that quercetin rescues the inhibitive effect of HuR that blocks the pri-miR-7–1 processing. Importantly, the quercetin-mediated dissociation of HuR/MSI2 with pri-miR-7–1 was confirmed by RP-SMS (RNA Pull-down SILAC Mass Spectrometry (37)) (Supplementary Figure S5D). Interestingly, luteolin had no significant effect on α-Syn or miR-7, suggesting different bioavailability, dynamics or cellular metabolism when compared with quercetin (Figure 8A–C).

To find out if quercetin acts through the miR-7/α-Syn pathway, we deleted the stem loop region of pri-miR-7–1 in the genome of HeLa cells, using CRISPR-Cas9 and generated miR-7–1^+/−^ and miR-7–1^−/−^ cell lines (Supplementary Figure S6). However, quercetin could still inhibit the expression of α-Syn, even though miR-7 was reduced by half or completely depleted (Figure 8D). Next, we tested these compounds in HuR KO HeLa cells generated by CRISPR-Cas9 (Supplementary Figure S2A,B,D). Importantly, in these cells, quercetin did not affect the levels of α-Syn expression (Figure 8E, F). Intriguingly, in HeLa HuR KO cells the miR-7 levels were upregulated by 2- and 3-fold by quercetin and luteolin, respectively (Figure 8G). This suggests more complex network of miRNA regulation in the absence of HuR. Finally, we investigated the mRNA levels of α-Syn after quercetin treatment. Quercetin significantly reduced α-Syn mRNA by 50% in wild-type HeLa and miR-7 KO cells, while a less significant 20% inhibition was seen in HuR KO HeLa cells (Figure 8H). Thus, we conclude that the HuR-mediated activity of quercetin towards α-Syn is exerted at least partially at the mRNA level. In summary, in our cellular model quercetin downregulates α-Syn expression and this regulation is HuR-dependent.

### Quercetin inhibits HuR binding to both pri-miR-7-1 and α-Syn mRNA in cells

**Figure 9.**
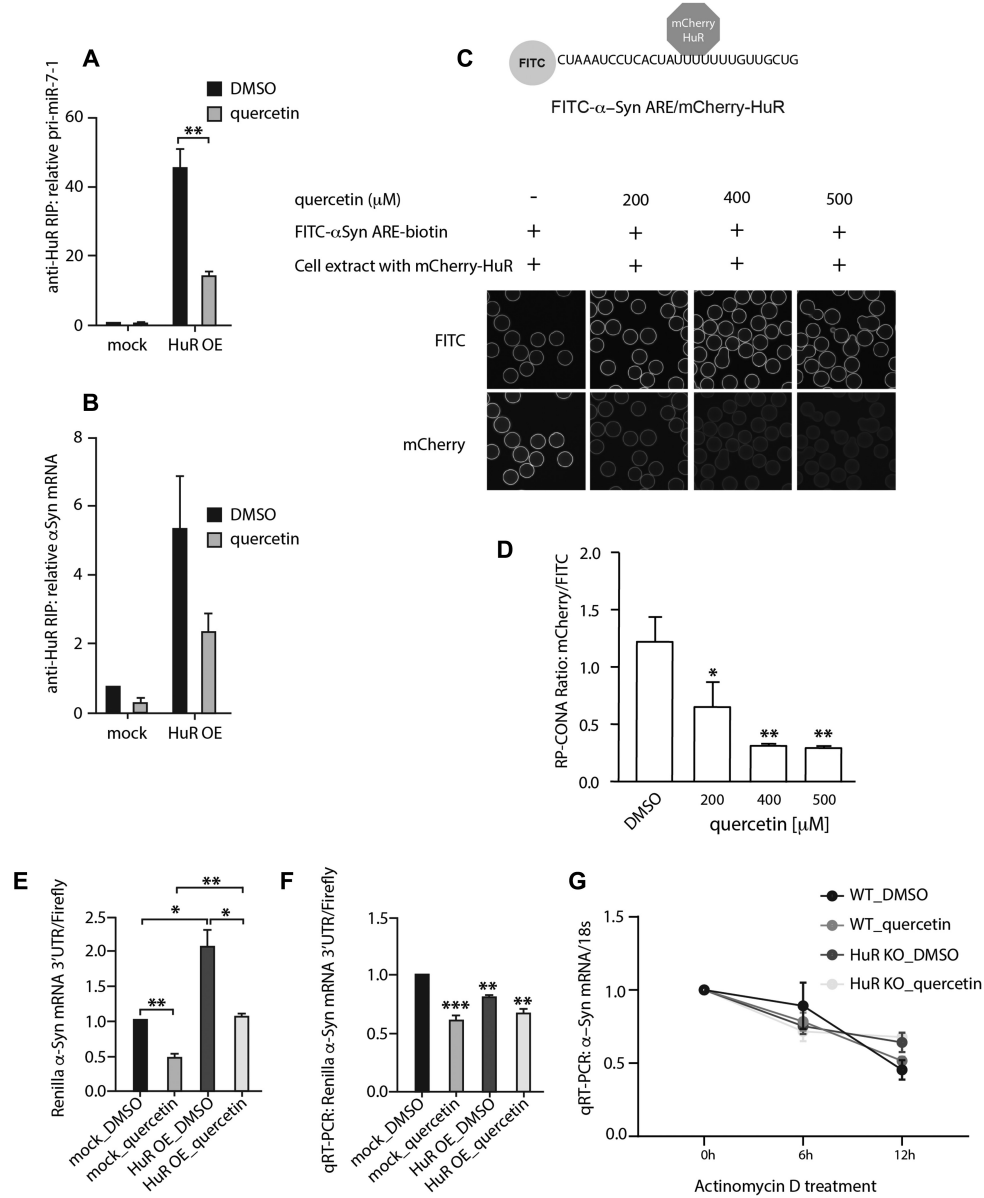
Quercetin interrupts HuR from binding to pri-miR-7–1 and α-Syn mRNA in cells. (**A** and **B**) Quercetin reduced efficiency of pri-miR-7–1 (A) and α-Syn mRNA (B) RIP with HuR in HeLa cells. Mock or HuR-overexpressed HuR KO HeLa cells were treated with DMSO or 20 μM of quercetin and harvested 48 h after treatment. HuR bound RNAs from cell lysates were immunoprecipitated by anti-HuR antibody-coated beads and quantified by qRT-PCR. RNA levels were normalized to DMSO treated mock samples. Mean relative RNA levels and SEM from three independent repeats are shown. Statistical tests were interpreted by SPSS independent sample *t*-test, ** *P*<0.01. (**C** and **D**). Quercetin inhibits α-Syn-ARE/HuR binding in RP-CONA. A diagram illustrates the interaction between FITC-α-Syn-ARE with mCherry-HuR. Cell extracts containing 50 nM of mCherry-HuR were pre-incubated with an increased concentration of quercetin, prior pull-down by FITC-α-Syn-ARE-beads. Mean mCherry/FITC ring intensities and SD between triplicates are shown. Statistically significant differences compared to DMSO were interpreted by SPSS independent sample *t*-test, * *P*< 0.05, **P< 0.01. (**E** and **F**). Quercetin inhibits the HuR-induced expression of luciferase bearing the 3′UTR of α-Syn mRNA. Mock or HuR overexpressed HeLa cells were treated with DMSO or 20 μM of quercetin, and transfected with the dual luciferase reporter carrying α-Syn-3′UTR downstream the Renilla luciferase gene. Mean luminescence levels (E) and mRNA levels (F) of Renilla/firefly luciferases, and SEM from 3 independent repeats are shown. Statistically significant differences between groups, or compared to mock DMSO were interpreted by SPSS independent sample *t*-test, *P< 0.05, **P< 0.01, ***P< 0.001. (**G**). The effects of quercetin on α-Syn mRNA decay. Wild-type and HuR KO HeLa cells were treated with DMSO or 20 μM of quercetin, supplemented with 10 μg/ml of actinomycin D for 0, 6 and 12 h, respectively. α-Syn mRNA and 18S levels were determined by qRT-PCR. Mean α-Syn/18S and SEM from three independent repeats are shown.

The 3′-UTR of α-Syn mRNA bears AU-rich elements (AREs) that are common binding targets of HuR (57). To further explore the quercetin/HuR/α-Syn pathway, we performed an RNA immunoprecipitation (RIP) assay on quercetin-treated HeLa cells with overexpressed HuR. The HuR-mediated immunoprecipitation of pri-miR-7–1 significantly decreased in the quercetin-treated cells (Figure 9A). We also observed a noticeable reduction of HuR-mediated immunoprecipitation of α-Syn mRNA upon quercetin treatment (Figure 9B). Therefore, quercetin inhibits HuR binding to both pri-miR-7–1 and α-Syn mRNA in living cells. We then validated the α-Syn-ARE/HuR binding in RP-CONA, and the interaction could be inhibited by quercetin at high concentrations (Figure 9C, D).

Finally, quercetin reduced luciferase levels in our dual luciferase reporter assay with α-Syn mRNA 3′-UTR coupled with Renilla luciferase (Figure 9E). The overexpression of HuR upregulated Renilla luciferase level by 2-fold, which was reduced to the mock control levels upon quercetin treatment. Notably, the relative levels of the Renilla luciferase mRNA were not enhanced by HuR (Figure 9F), implying HuR induces the translation of α-Syn through the 3′-UTR, and this can be reversed by quercetin. In the stability assay, quercetin treatment only slightly destabilized the α-Syn mRNA in WT HeLa cells after actinomycin D treatment for 6 h, while exerted no effects in the HuR KO cells (Figure 9G). In conclusion, quercetin interrupts the α-Syn-ARE/HuR interaction, suppressing the HuR-engaged α-Syn expression mainly at the translational level with some contribution from regulating mRNA levels. However, the contribution of quercetin-mediated induction of miR-7 towards destabilization of α-Syn mRNA could be still important in other cellular systems such as mature neurones.

## DISCUSSION

α-Syn has become a popular target for investigational PD therapies, with RNA interference (RNAi) strategies focusing on α-Syn repression presently at preclinical stages (58). An shRNA therapeutic achieved a 35% knockdown of SNpc α-Syn, and showed protective effects in a PD rat model without notable toxicity (59). However, neuronal toxicity has been found in animal brains when another siRNA-induced α-Syn knockdown reached 90% (60,61). Therefore, the knockdown efficiency of α-Syn-targeted RNAi needs to be tightly controlled within an appropriate range in order to reach a compromise of both safety and efficacy (61,62). MiRNAs, known as fine tuners of gene expression, buffer the expression network against environmental or genetic stress (63). Here we provide evidence that miR-7 is the most effective suppressor of α-Syn expression, among all potential α-Syn targeting miRs that are downregulated in PD (Figure 1). Therefore, fine-tuning α-Syn expression by elevating miR-7 level could provide a novel avenue for the development of PD therapy.

Previously we have shown that miR-7 biogenesis is inhibited through an interaction between the pri-miR-7–1-CTL and HuR (32). The best elucidated miR post-transcriptional regulation is between let-7 and Lin28 proteins. A range of screens targeting let-7/Lin28 have been carried out using fluorescence resonance energy transfer (FRET) or fluorescence polarization (FP) methods, and some hits have shown promising anti-cancer properties by dissociating the RNA–protein interaction and upregulating let-7 levels (6). However, due to a lack of understanding of the interaction domains where pri-miR-7 binds HuR, it seems challenging to identify pri-miR-7–1/HuR inhibitors using similar approaches. To tackle this problem, we developed the RP-CONA technique that combines the RNA pull-down assay in human cell extracts with the CONA screening platform (37–39).

The RNA pull-down assay captures proteins by on-bead RNA fragments from eukaryotic cell lysates (37). The application of cell extracts instead of purified protein allows the screening to take place in a more physiological environment and might help avoiding false positives that are not functional *in vivo*. Moreover, this technique removes the need of protein purification, making it an alternative solution to study RNA/protein binding events for those proteins with difficulty in purification. CONA is a previously established, sensitive and quantifiable on-bead imaging platform (64,65). The only known ligand targeting the RNA recognition motif-3 (RRM3) of HuR was identified by CONA from one-bead-one-compound libraries (66). Notably, using the RRM3 binder and CONA, an ATP-binding pocket was discovered in RRM3, which was not detected using conventional binding assays (66,67). CONA is also highly flexible, and it can be modified to monitor complex biological processes in real time, such as ubiquitination-related enzymatic activities and aggregation of α-Syn (38,40). A recent publication successfully identified protein binders from natural product extracts with CONA (41). Here, for the first time we applied CONA to study RNA–protein interactions by using a lysate-based screening strategy. We believe that this combination has a high potential for further exploitation in miniaturized drug screening. RP-CONA is a versatile technique that is not limited to monitor the pri-miR-7/HuR activity. We have shown that RP-CONA is applicable in different RNA/protein complexes such as pre-let-7a-1/Lin28a (Figure 5), αSyn-ARE/HuR (Figure 9C) and TNFα-ARE/HuR (Supplementary Figure S7). This provides an alternative method to look for RNA-protein modulators in the environment of the mammalian cell extracts.

In order to look for pri-miR-7–1/HuR inhibitors, we started with a focused prototype screen. The library contained eight compounds that were known to interfere with the RNA binding activities of HuR or MSI2, as both of the proteins are essential during the blockage of miR-7 biogenesis (6). We found half of the compounds resulting in >50% inhibition of RP-CONA signals (Figure 6). By integrating the focused library with a diverse in-house 54-compound library, we have shown that the former 4 compounds were still effective, and only one additional hit was discovered from the new library. The disruptive effects of these hits were confirmed in RP-CONA with a gradient of concentrations, and the IC_50_s of the most effective hits quercetin and luteolin were around 2 μM. We subsequently validated the primary hits using the standard RNA pull-down assay followed by western blot and managed to exclude the unspecific disruptor gossypol, which also interrupted the binding of the reference protein DHX9 (Figure 7). Previously, quercetin was recognized as a pre-let-7g/Lin28a inhibitor with <2.5 μM IC50s in EMSA and a fluorescence intensity‐based binding assay (68). However, in our hands 100 μM of quercetin only mildly inhibited Lin28a binding to pre-let-7a-1-CTL in RP-CONA (Supplementary Figure S8), suggesting that quercetin is more effective towards pri-miR-7–1/HuR in cell extracts.

Quercetin has been shown to inhibit the interaction between HuR and TNF-α mRNA, with an IC_50_ of 1.4 μM in RNA electrophoretic mobility gel shift assay (EMSA) (42). Luteolin binds the RRM1 of MSI1, the paralogue of MSI2, with a dissociation constant (*K*_d_) of around 3 μM (69). Interestingly, luteolin is a close analogue of quercetin. In an FP assay, both luteolin and quercetin interrupted MSI1-RRM1 binding with a short RNA motif at micromolar and millimolar IC_50_s, respectively (69). These findings imply that quercetin is likely to be a dual-inhibitor of both HuR and MSI2, marking it a strong candidate as an miR-7 enhancer. As expected, we identified a 1.5-fold upregulation of mature miR-7 in HeLa cells after 20 μM of quercetin treatment (Figure 8C). This is consistent with what we saw when HuR or MSI2 or both were knocked down (32). At the same time, a significant downregulation of α-Syn expression was observed (Figure 8A, B). It has been reported that quercetin exerts neuroprotective effects in different cell or animal models of PD (70–72). Moreover, oxidized quercetin can prevent α-Syn fibrillization *in vitro* (73). Interestingly, in neuron-like PC12 cells α-Syn expression was induced by 50–500 μM of quercetin, albeit reduced when quercetin reached 1 mM (74). These discrepancies may be attributed to the selection of cell lines. We also used quercetin to treat human neuroblastoma SH-SY5Y and neuron-like mouse embryonic carcinoma P19 cell lines. The reduction of α-Syn expression was notable but was less significant than that observed in HeLa cells (Supplementary Figure S5E). As mentioned above, HeLa cells were selected due to its abundant expression of α-Syn, so the reduced α-Syn level could have been easily detected. Additionally, luteolin did not show similar effects of miR-7 induction or α-Syn inhibition as its analogue quercetin (Figure 8A–C). This suggests quercetin and luteolin may present different bioavailability in living cells, despite their similar performance in RP-CONA. This is reminiscent of the evidence that quercetin was readily incorporated into cells, while another close flavonoid analogue myrincetin had poor cellular uptake (75). Collectively, we present the first report of quercetin as a miR-7 enhancer and α-Syn inhibitor at low concentrations.

We hypothesized that quercetin inhibits α-Syn through the pri-miR-7–1/HuR pathway. However, we found similar inhibitive effects of quercetin in miR-7 KO HeLa cells, which means the upregulated miR-7 is not the major contributor of α-Syn suppression (Figure 8D). Indeed, quercetin has reduced α-Syn expression by around 60% in wild-type HeLa cells, and it would require >1000-fold miR-7 upregulation to achieve a similar extent of α-Syn inhibition in the same cell line (Figure 1, Supplementary Figure S1A). Crucially, we found that α-Syn remained unchanged upon quercetin treatment when HuR was absent (Figure 8E, F). Nevertheless, the upregulation of miR-7 was still observed (Figure 8G). These results strongly imply that HuR is an essential part in the quercetin-mediated inhibition of α-Syn levels, although it seems not connected to the regulation of miR-7 biogenesis in our cellular system. Moreover, the biogenesis of miR-7 is also regulated by some other protein complexes, including SF2/ASF, NF45-NF90 and QKI (76–78). Therefore, the removal of HuR may allow alternative miR-7 regulation pathways to take over. This may explain why luteolin did not affect miR-7 level in wild-type HeLa, but significantly induced miR-7 in HuR KO HeLa cells.

HuR is known to stabilize target mRNAs through the AREs in their 3′-UTRs (57). A recent study indicates that HuR interacts with the 3′-UTR of α-Syn mRNA and increases its stability in an mRNA decay assay in HeLa cells, although the knockdown of HuR only shows mild inhibition on α-Syn expression. Importantly, the stabilization seems to be independent of miR-7 (79). In our study, quercetin decreased α-Syn mRNA, and it was HuR-dependent. At the same time, in HeLa cells, the pri-miR-7–1/HuR pathway is not a major contributor of quercetin-mediated α-Syn inhibition (Figure 8H). Using an immunoprecipitation assay, we found that quercetin inhibited HuR from interacting with pri-miR-7–1 in cells, and the effect towards α-Syn mRNA was milder (Figure 9A). Consistently, in RP-CONA, quercetin was effective at 20 μM on inhibiting pri-miR-7–1-CTL/HuR (Figure 7D), but it required 200 μM of quercetin to interrupt α-Syn-ARE/HuR (Figure 9D), as well as TNFα-ARE/HuR (Supplementary Figure S7). This suggests higher affinity between the α-Syn-ARE/HuR interaction than pri-miR-7–1-CTL/HuR, or different acting mechanisms of quercetin. Our luciferase assays showed that the overexpressed HuR enhanced the expression of luciferase with α-Syn mRNA 3′-UTR at translational level, which could be reversed by quercetin (Figure 9E, F). In an actinomycin D-based stability assay, endogenous HuR only slightly enhanced the stability of α-Syn mRNA, which could be eliminated by quercetin (Figure 9G). However, this assay may not be the best model to study the effects of quercetin on α-Syn mRNA stability, and the treatment time of 12 h (limited by actinomycin D toxicity) could be too short to induce significant effects. As there is clear evidence of quercetin reducing α-Syn mRNA levels after 48-h treatment (Figure 8H), we cannot rule out the possibility that quercetin reduces steady state levels of α-Syn mRNA by regulating other post-transcriptional processes including splicing, export or localization. These assumptions are supported by some evidences with HuR regulating mRNA alternative splicing (80), as well as alternative polyadenylation (81). Based on our results we conclude that quercetin interrupts HuR binding with both pri-miR-7–1 and α-Syn mRNA, promoting miR-7 biogenesis, inhibiting α-Syn translation and reducing α-Syn transcripts levels. All of these together contribute to the strong repression on α-Syn expression by quercetin.

To summarize, we developed an on-bead screening platform, RP-CONA, for the identification of RNA–protein interaction modulators. We employed RP-CONA to look for inhibitors of pri-miR-7–1/HuR interactions. The most potent hit quercetin was proven to be a miR-7 enhancer as well as an α-Syn inhibitor, implying a potential as PD therapeutic. The mechanism of quercetin-mediated inhibition of α-Syn is HuR-dependent. Additionally, quercetin displayed positive effects on miR-7 levels, but the achieved upregulation was too small to directly affect α-Syn. Further work needs to be focused on the evaluation of quercetin in PD models, especially in the context of mature neurones as well as α-Syn overproduction and accumulation. In the meantime, the RP-CONA screening method can be used to identify more potent and specific miR-7 enhancers and α-Syn inhibitors. In summary, our work delivers a new platform for the identification of RNA-protein interaction modulators, and highlights quercetin as a potential hit compound towards treatment of PD.

## DATA AVALIABILITY

All data generated or analysed during this study are included in this published article (and its additional files). The mass spectrometry proteomics data have been deposited in the ProteomeXchange Consortium via the PRIDE (82) partner repository with the dataset identifier PXD025617. Requests for material should be made to the corresponding author.

## Supplementary Material

gkab484_Supplemental_File

## References

[1] García-Mauriño S.M. , Rivero-Rodríguez F., Velázquez-Cruz A., Hernández-Vellisca M., Díaz-Quintana A., De la Rosa M.A., Díaz-Moreno I. RNA binding protein regulation and cross-talk in the control of AU-rich mRNA fate. Front. Mol. Biosci. 2017; 4:71. 29109951 10.3389/fmolb.2017.00071 PMC5660096

[2] Obeng E.A. , Stewart C., Abdel-Wahab O. Altered RNA processing in cancer pathogenesis and therapy. Cancer Discov. 2019; 9:1493–1510. 31611195 10.1158/2159-8290.CD-19-0399 PMC6825565

[3] Nussbacher J.K. , Tabet R., Yeo G.W., Lagier-Tourenne C. Disruption of RNA metabolism in neurological diseases and emerging therapeutic interventions. Neuron. 2019; 102:294–320. 30998900 10.1016/j.neuron.2019.03.014 PMC6545120

[4] Dhillon S. Risdiplam: first approval. Drugs. 2020; 80:1853–1858. 33044711 10.1007/s40265-020-01410-z

[5] Campagne S. , Boigner S., Rüdisser S., Moursy A., Gillioz L., Knörlein A., Hall J., Ratni H., Cléry A., Allain F.H. Structural basis of a small molecule targeting RNA for a specific splicing correction. Nat. Chem. Biol. 2019; 15:1191–1198. 31636429 10.1038/s41589-019-0384-5 PMC7617061

[6] Zhu S. , Rooney S., Michlewski G. RNA-targeted therapies and high-throughput screening methods. Int. J. Mol. Sci. 2020; 21:2996. 10.3390/ijms21082996 PMC7216119 32340368

[7] Mitchell P.S. , Parkin R.K., Kroh E.M., Fritz B.R., Wyman S.K., Pogosova-Agadjanyan E.L., Peterson A., Noteboom J., O’Briant K.C., Allen A. et al. Circulating microRNAs as stable blood-based markers for cancer detection. Proceed. Natl. Acad. Sci. USA. 2008; 105:10513–10518. 10.1073/pnas.0804549105 PMC2492472 18663219

[8] Rupaimoole R. , Slack F.J. MicroRNA therapeutics: towards a new era for the management of cancer and other diseases. Nat. Rev. Drug Discov. 2017; 16:203–222. 28209991 10.1038/nrd.2016.246

[9] Landthaler M. , Yalcin A., Tuschl T. The human DiGeorge syndrome critical region gene 8 and Its D. melanogaster homolog are required for miRNA biogenesis. Curr. Biol. 2004; 14:2162–2167. 15589161 10.1016/j.cub.2004.11.001

[10] Gregory R.I. , Yan K.P., Amuthan G., Chendrimada T., Doratotaj B., Cooch N., Shiekhattar R. The Microprocessor complex mediates the genesis of microRNAs. Nature. 2004; 432:235–240. 15531877 10.1038/nature03120

[11] Han J. , Lee Y., Yeom K.H., Kim Y.K., Jin H., Kim V.N. The Drosha-DGCR8 complex in primary microRNA processing. Genes Dev. 2004; 18:3016–3027. 15574589 10.1101/gad.1262504 PMC535913

[12] Denli A.M. , Tops B.B., Plasterk R.H., Ketting R.F., Hannon G.J. Processing of primary microRNAs by the microprocessor complex. Nature. 2004; 432:231–235. 15531879 10.1038/nature03049

[13] Nguyen T.A. , Jo M.H., Choi Y.G., Park J., Kwon S.C., Hohng S., Kim V.N., Woo J.S. Functional anatomy of the human microprocessor. Cell. 2015; 161:1374–1387. 26027739 10.1016/j.cell.2015.05.010

[14] Hutvágner G. , McLachlan J., Pasquinelli A.E., Bálint E., Tuschl T., Zamore P.D. A cellular function for the RNA-interference enzyme Dicer in the maturation of the let-7 small temporal RNA. Science. 2001; 293:834–838. 11452083 10.1126/science.1062961

[15] Ketting R.F. , Fischer S.E., Bernstein E., Sijen T., Hannon G.J., Plasterk R.H. Dicer functions in RNA interference and in synthesis of small RNA involved in developmental timing in C. elegans. Genes Dev. 2001; 15:2654–2659. 11641272 10.1101/gad.927801 PMC312808

[16] Krol J. , Loedige I., Filipowicz W. The widespread regulation of microRNA biogenesis, function and decay. Nat. Rev. Genet. 2010; 11:597–610. 20661255 10.1038/nrg2843

[17] Reeve A. , Simcox E., Turnbull D. Ageing and Parkinson’s disease: why is advancing age the biggest risk factor. Ageing Res. Rev. 2014; 14:19–30. 24503004 10.1016/j.arr.2014.01.004 PMC3989046

[18] Lesage S. , Brice A. Parkinson’s disease: from monogenic forms to genetic susceptibility factors. Hum. Mol. Genet. 2009; 18:R48–R59. 19297401 10.1093/hmg/ddp012

[19] Kalia L.V. , Lang A.E. Parkinson’s disease. Lancet. 2015; 386:896–912. 25904081 10.1016/S0140-6736(14)61393-3

[20] Poewe W. , Seppi K., Tanner C.M., Halliday G.M., Brundin P., Volkmann J., Schrag A.E., Lang A.E. Parkinson disease. Nat. Rev. Dis. Primers. 2017; 3:17013. 28332488 10.1038/nrdp.2017.13

[21] Spillantini M.G. , Schmidt M.L., Lee V.M., Trojanowski J.Q., Jakes R., Goedert M. Alpha-synuclein in Lewy bodies. Nature. 1997; 388:839–840. 9278044 10.1038/42166

[22] Dehay B. , Bourdenx M., Gorry P., Przedborski S., Vila M., Hunot S., Singleton A., Olanow C.W., Merchant K.M., Bezard E. et al. Targeting alpha-synuclein for treatment of Parkinson's disease: mechanistic and therapeutic considerations. Lancet Neurol. 2015; 14:855–866. 26050140 10.1016/S1474-4422(15)00006-X PMC5217462

[23] Junn E. , Lee K.W., Jeong B.S., Chan T.W., Im J.Y., Mouradian M.M. Repression of alpha-synuclein expression and toxicity by microRNA-7. Proc. Natl. Acad. Sci. USA. 2009; 106:13052–13057. 19628698 10.1073/pnas.0906277106 PMC2722353

[24] McMillan K.J. , Murray T.K., Bengoa-Vergniory N., Cordero-Llana O., Cooper J., Buckley A., Wade-Martins R., Uney J.B., O’Neill M.J., Wong L.F. et al. Loss of microRNA-7 regulation leads to α-synuclein accumulation and dopaminergic neuronal loss in vivo. Mol. Ther. 2017; 25:2404–2414. 28927576 10.1016/j.ymthe.2017.08.017 PMC5628933

[25] Zhao L. , Wang Z. MicroRNAs: game changers in the regulation of alpha-synuclein in Parkinson’s disease. Parkinsons Dis. 2019; 2019:1743183. 31191899 10.1155/2019/1743183 PMC6525811

[26] Titze-de-Almeida R. , Titze-de-Almeida S.S. miR-7 replacement therapy in Parkinson’s disease. Curr. Gene Ther. 2018; 18:143–153. 29714132 10.2174/1566523218666180430121323

[27] Michlewski G. , Caceres J.F. Post-transcriptional control of miRNA biogenesis. RNA. 2019; 25:1–16. 30333195 10.1261/rna.068692.118 PMC6298569

[28] Treiber T. , Treiber N., Meister G. Regulation of microRNA biogenesis and its crosstalk with other cellular pathways. Nat. Rev. Mol. Cell Biol. 2019; 20:5–20. 30728477 10.1038/s41580-019-0106-6

[29] Creugny A. , Fender A., Pfeffer S. Regulation of primary microRNA processing. FEBS Lett. 2018; 592:1980–1996. 29683487 10.1002/1873-3468.13067

[30] Towbin H. , Wenter P., Guennewig B., Imig J., Zagalak J.A., Gerber A.P., Hall J. Systematic screens of proteins binding to synthetic microRNA precursors. Nucleic Acids Res. 2013; 41:e47. 23221640 10.1093/nar/gks1197 PMC3561989

[31] Michlewski G. , Guil S., Semple C.A., Caceres J.F. Posttranscriptional regulation of miRNAs harboring conserved terminal loops. Mol. Cell. 2008; 32:383–393. 18995836 10.1016/j.molcel.2008.10.013 PMC2631628

[32] Choudhury N.R. , de Lima Alves F., de Andres-Aguayo L., Graf T., Caceres J.F., Rappsilber J., Michlewski G. Tissue-specific control of brain-enriched miR-7 biogenesis. Genes Dev. 2013; 27:24–38. 23307866 10.1101/gad.199190.112 PMC3553281

[33] Lebedeva S. , Jens M., Theil K., Schwanhausser B., Selbach M., Landthaler M., Rajewsky N. Transcriptome-wide analysis of regulatory interactions of the RNA-binding protein HuR. Mol. Cell. 2011; 43:340–352. 21723171 10.1016/j.molcel.2011.06.008

[34] Clingman C.C. , Deveau L.M., Hay S.A., Genga R.M., Shandilya S.M., Massi F., Ryder S.P. Allosteric inhibition of a stem cell RNA-binding protein by an intermediary metabolite. Elife. 2014; 3:e02848. 10.7554/eLife.02848 PMC4094780 24935936

[35] Kumar S. , Downie Ruiz Velasco A., Michlewski G. Oleic acid induces MiR-7 processing through remodeling of Pri-MiR-7/protein complex. J. Mol. Biol. 2017; 429:1638–1649. 28483648 10.1016/j.jmb.2017.05.001 PMC5462424

[36] Cury-Boaventura M.F. , Pompeia C., Curi R. Comparative toxicity of oleic acid and linoleic acid on Jurkat cells. Clin. Nutr. 2004; 23:721–732. 15297111 10.1016/j.clnu.2003.12.004

[37] Choudhury N.R. , Michlewski G. Quantitative identification of proteins that influence miRNA biogenesis by RNA pull-down-SILAC mass spectrometry (RP-SMS). Methods. 2019; 152:12–17. 29890283 10.1016/j.ymeth.2018.06.006 PMC6335501

[38] Koszela J. , Pham N.T., Evans D., Mann S., Perez-Pi I., Shave S., Ceccarelli D.F.J., Sicheri F., Tyers M., Auer M. Real-time tracking of complex ubiquitination cascades using a fluorescent confocal on-bead assay. BMC Biol. 2018; 16:88. 30097011 10.1186/s12915-018-0554-z PMC6086040

[39] Hintersteiner M. , Ambrus G., Bednenko J., Schmied M., Knox A.J., Meisner N.C., Gstach H., Seifert J.M., Singer E.L., Gerace L. et al. Identification of a small molecule inhibitor of importin beta mediated nuclear import by confocal on-bead screening of tagged one-bead one-compound libraries. ACS Chem. Biol. 2010; 5:967–979. 20677820 10.1021/cb100094k PMC2956136

[40] Pérez-Pi I. , Evans D.A., Horrocks M.H., Pham N.T., Dolt K.S., Koszela J., Kunath T., Auer M. ASYN-CONA, a novel bead-based assay for detecting early stage α-synuclein aggregation. Anal. Chem. 2019; 91:5582–5590. 30964656 10.1021/acs.analchem.8b03842 PMC6534341

[41] Ambrose A.J. , Pham N.T., Sivinski J., Guimarães L., Mollasalehi N., Jimenez P., Abad M.A., Jeyaprakash A.A., Shave S., Costa-Lotufo L.V. et al. A two-step resin based approach to reveal survivin-selective fluorescent probes. RSC Chem. Biol. 2021; 2:181–186. 10.1039/d0cb00122h PMC8342005 34458780

[42] Chae M.J. , Sung H.Y., Kim E.H., Lee M., Kwak H., Chae C.H., Kim S., Park W.Y. Chemical inhibitors destabilize HuR binding to the AU-rich element of TNF-alpha mRNA. Exp. Mol. Med. 2009; 41:824–831. 19949288 10.3858/emm.2009.41.11.088 PMC2788736

[43] Zhang J.H. , Chung T.D., Oldenburg K.R. A simple statistical parameter for use in evaluation and validation of high throughput screening assays. J. Biomol. Screen. 1999; 4:67–73. 10838414 10.1177/108705719900400206

[44] Nowak J.S. , Choudhury N.R., de Lima Alves F., Rappsilber J., Michlewski G. Lin28a regulates neuronal differentiation and controls miR-9 production. Nat. Commun. 2014; 5:3687. 24722317 10.1038/ncomms4687 PMC4035284

[45] Niranjanakumari S. , Lasda E., Brazas R., Garcia-Blanco M.A. Reversible cross-linking combined with immunoprecipitation to study RNA-protein interactions in vivo. Methods. 2002; 26:182–190. 12054895 10.1016/S1046-2023(02)00021-X

[46] Schulz J. , Takousis P., Wohlers I., Itua I.O.G., Dobricic V., Rucker G., Binder H., Middleton L., Ioannidis J.P.A., Perneczky R. et al. Meta-analyses identify differentially expressed micrornas in Parkinson’s disease. Ann. Neurol. 2019; 85:835–851. 30990912 10.1002/ana.25490

[47] Agarwal V. , Bell G.W., Nam J.W., Bartel D.P. Predicting effective microRNA target sites in mammalian mRNAs. Elife. 2015; 4:e05005. 10.7554/eLife.05005 PMC4532895 26267216

[48] Chou C.H. , Shrestha S., Yang C.D., Chang N.W., Lin Y.L., Liao K.W., Huang W.C., Sun T.H., Tu S.J., Lee W.H. et al. miRTarBase update 2018: a resource for experimentally validated microRNA-target interactions. Nucleic Acids Res. 2018; 46:D296–D302. 29126174 10.1093/nar/gkx1067 PMC5753222

[49] Uhlen M. , Zhang C., Lee S., Sjöstedt E., Fagerberg L., Bidkhori G., Benfeitas R., Arif M., Liu Z., Edfors F. et al. A pathology atlas of the human cancer transcriptome. Science. 2017; 357:eaan2507. 28818916 10.1126/science.aan2507

[50] Doxakis E. Post-transcriptional regulation of alpha-synuclein expression by mir-7 and mir-153. J. Biol. Chem. 2010; 285:12726–12734. 20106983 10.1074/jbc.M109.086827 PMC2857101

[51] Niu M. , Xu R., Wang J., Hou B., Xie A. MiR-133b ameliorates axon degeneration induced by MPP(+) via targeting RhoA. Neuroscience. 2016; 325:39–49. 27012608 10.1016/j.neuroscience.2016.03.042

[52] Cazalla D. , Sanford J.R., Caceres J.F. A rapid and efficient protocol to purify biologically active recombinant proteins from mammalian cells. Protein Expr. Purif. 2005; 42:54–58. 15878828 10.1016/j.pep.2005.03.035

[53] Heo I. , Joo C., Cho J., Ha M., Han J., Kim V.N. Lin28 mediates the terminal uridylation of let-7 precursor MicroRNA. Mol. Cell. 2008; 32:276–284. 18951094 10.1016/j.molcel.2008.09.014

[54] Newman M.A. , Thomson J.M., Hammond S.M. Lin-28 interaction with the Let-7 precursor loop mediates regulated microRNA processing. RNA. 2008; 14:1539–1549. 18566191 10.1261/rna.1155108 PMC2491462

[55] Viswanathan S.R. , Daley G.Q., Gregory R.I. Selective blockade of microRNA processing by Lin28. Science. 2008; 320:97–100. 18292307 10.1126/science.1154040 PMC3368499

[56] Michlewski G. , Caceres J.F. RNase-assisted RNA chromatography. RNA. 2010; 16:1673–1678. 20571124 10.1261/rna.2136010 PMC2905764

[57] Schultz C.W. , Preet R., Dhir T., Dixon D.A., Brody J.R. Understanding and targeting the disease-related RNA binding protein human antigen R (HuR). Wiley Interdiscip Rev RNA. 2020; 11:e1581. 31970930 10.1002/wrna.1581 PMC7482136

[58] Teil M. , Arotcarena M.L., Faggiani E., Laferriere F., Bezard E., Dehay B. Targeting α-synuclein for PD therapeutics: a pursuit on all fronts. Biomolecules. 2020; 10:391. 10.3390/biom10030391 PMC7175302 32138193

[59] Zharikov A.D. , Cannon J.R., Tapias V., Bai Q., Horowitz M.P., Shah V., El Ayadi A., Hastings T.G., Greenamyre J.T., Burton E.A. shRNA targeting α-synuclein prevents neurodegeneration in a Parkinson’s disease model. J. Clin. Invest. 2015; 125:2721–2735. 26075822 10.1172/JCI64502 PMC4563670

[60] Gorbatyuk O.S. , Li S., Nash K., Gorbatyuk M., Lewin A.S., Sullivan L.F., Mandel R.J., Chen W., Meyers C., Manfredsson F.P. et al. In vivo RNAi-mediated alpha-synuclein silencing induces nigrostriatal degeneration. Mol. Ther. 2010; 18:1450–1457. 20551914 10.1038/mt.2010.115 PMC2927065

[61] Collier T.J. , Redmond D.E., Steece-Collier K., Lipton J.W., Manfredsson F.P. Is alpha-synuclein loss-of-function a contributor to Parkinsonian pathology? Evidence from non-human primates. Front. Neurosci. 2016; 10:12. 26858591 10.3389/fnins.2016.00012 PMC4731516

[62] Brundin P. , Dave K.D., Kordower J.H. Therapeutic approaches to target alpha-synuclein pathology. Exp. Neurol. 2017; 298:225–235. 28987463 10.1016/j.expneurol.2017.10.003 PMC6541231

[63] Vidigal J.A. , Ventura A. The biological functions of miRNAs: lessons from in vivo studies. Trends Cell Biol. 2015; 25:137–147. 25484347 10.1016/j.tcb.2014.11.004 PMC4344861

[64] Hintersteiner M. , Buehler C., Uhl V., Schmied M., Muller J., Kottig K., Auer M. Confocal nanoscanning, bead picking (CONA): PickoScreen microscopes for automated and quantitative screening of one-bead one-compound libraries. J. Comb. Chem. 2009; 11:886–894. 19603813 10.1021/cc900059q

[65] Hintersteiner M. , Kimmerlin T., Kalthoff F., Stoeckli M., Garavel G., Seifert J.M., Meisner N.C., Uhl V., Buehler C., Weidemann T. et al. Single bead labeling method for combining confocal fluorescence on-bead screening and solution validation of tagged one-bead one-compound libraries. Chem. Biol. 2009; 16:724–735. 19635409 10.1016/j.chembiol.2009.06.011

[66] Meisner N.C. , Hintersteiner M., Seifert J.M., Bauer R., Benoit R.M., Widmer A., Schindler T., Uhl V., Lang M., Gstach H. et al. Terminal adenosyl transferase activity of posttranscriptional regulator HuR revealed by confocal on-bead screening. J. Mol. Biol. 2009; 386:435–450. 19109971 10.1016/j.jmb.2008.12.020

[67] Ripin N. , Boudet J., Duszczyk M.M., Hinniger A., Faller M., Krepl M., Gadi A., Schneider R.J., Šponer J., Meisner-Kober N.C. et al. Molecular basis for AU-rich element recognition and dimerization by the HuR C-terminal RRM. Proc. Natl. Acad. Sci. USA. 2019; 116:2935–2944. 30718402 10.1073/pnas.1808696116 PMC6386705

[68] Byun W.G. , Lim D., Park S.B. Discovery of small-molecule modulators of protein-RNA interactions by fluorescence intensity-based binding assay. ChemBioChem. 2019; 21:818–824. 31587454 10.1002/cbic.201900467

[69] Yi C. , Li G., Ivanov D.N., Wang Z., Velasco M.X., Hernández G., Kaundal S., Villarreal J., Gupta Y.K., Qiao M. et al. Luteolin inhibits Musashi1 binding to RNA and disrupts cancer phenotypes in glioblastoma cells. RNA Biol. 2018; 15:1420–1432. 30362859 10.1080/15476286.2018.1539607 PMC6284574

[70] Zhang Z.J. , Cheang L.C., Wang M.W., Lee S.M. Quercetin exerts a neuroprotective effect through inhibition of the iNOS/NO system and pro-inflammation gene expression in PC12 cells and in zebrafish. Int. J. Mol. Med. 2011; 27:195–203. 21132259 10.3892/ijmm.2010.571

[71] Karuppagounder S.S. , Madathil S.K., Pandey M., Haobam R., Rajamma U., Mohanakumar K.P. Quercetin up-regulates mitochondrial complex-I activity to protect against programmed cell death in rotenone model of Parkinson's disease in rats. Neuroscience. 2013; 236:136–148. 23357119 10.1016/j.neuroscience.2013.01.032

[72] Ay M. , Luo J., Langley M., Jin H., Anantharam V., Kanthasamy A., Kanthasamy A.G. Molecular mechanisms underlying protective effects of quercetin against mitochondrial dysfunction and progressive dopaminergic neurodegeneration in cell culture and MitoPark transgenic mouse models of Parkinson's Disease. J. Neurochem. 2017; 141:766–782. 28376279 10.1111/jnc.14033 PMC5643047

[73] Zhu M. , Han S., Fink A.L. Oxidized quercetin inhibits α-synuclein fibrillization. Biochim. Biophys. Acta. 2013; 1830:2872–2881. 23295967 10.1016/j.bbagen.2012.12.027

[74] Ahn T.B. , Jeon B.S. The role of quercetin on the survival of neuron-like PC12 cells and the expression of α-synuclein. Neural Regen. Res. 2015; 10:1113–1119. 26330835 10.4103/1673-5374.160106 PMC4541243

[75] Drummond N.J. , Davies N.O., Lovett J.E., Miller M.R., Cook G., Becker T., Becker C.G., McPhail D.B., Kunath T. A synthetic cell permeable antioxidant protects neurons against acute oxidative stress. Sci. Rep. 2017; 7:11857. 28928373 10.1038/s41598-017-12072-5 PMC5605738

[76] Wu H. , Sun S., Tu K., Gao Y., Xie B., Krainer A.R., Zhu J. A splicing-independent function of SF2/ASF in microRNA processing. Mol. Cell. 2010; 38:67–77. 20385090 10.1016/j.molcel.2010.02.021 PMC3395997

[77] Higuchi T. , Todaka H., Sugiyama Y., Ono M., Tamaki N., Hatano E., Takezaki Y., Hanazaki K., Miwa T., Lai S. et al. Suppression of microRNA-7 (miR-7) biogenesis by nuclear factor 90-nuclear factor 45 complex (NF90-NF45) controls cell proliferation in hepatocellular carcinoma. J. Biol. Chem. 2016; 291:21074–21084. 27519414 10.1074/jbc.M116.748210 PMC5076517

[78] Wang Y. , Vogel G., Yu Z., Richard S. The QKI-5 and QKI-6 RNA binding proteins regulate the expression of microRNA 7 in glial cells. Mol. Cell. Biol. 2013; 33:1233–1243. 23319046 10.1128/MCB.01604-12 PMC3592017

[79] Marchese D. , Botta-Orfila T., Cirillo D., Rodriguez J.A., Livi C.M., Fernández-Santiago R., Ezquerra M., Martí M.J., Bechara E., Tartaglia G.G. et al. Discovering the 3' UTR-mediated regulation of alpha-synuclein. Nucleic Acids Res. 2017; 45:12888–12903. 29149290 10.1093/nar/gkx1048 PMC5728410

[80] Lee S. , Wei L., Zhang B., Goering R., Majumdar S., Wen J., Taliaferro J.M., Lai E.C. ELAV/Hu RNA binding proteins determine multiple programs of neural alternative splicing. PLoS Genet. 2021; 17:e1009439. 33826609 10.1371/journal.pgen.1009439 PMC8055025

[81] Pereira-Castro I. , Moreira A. On the function and relevance of alternative 3'-UTRs in gene expression regulation. Wiley Interdiscip. Rev. RNA. 2021; 2021:e1653. 10.1002/wrna.1653 33843145

[82] Vizcaino J.A. , Csordas A., del-Toro N., Dianes J.A., Griss J., Lavidas I., Mayer G., Perez-Riverol Y., Reisinger F., Ternent T. et al. 2016 update of the PRIDE database and its related tools. Nucleic Acids Res. 2016; 44:D447–D456. 26527722 10.1093/nar/gkv1145 PMC4702828

