# Supplementary material for: RNA pull-down confocal nanoscanning (RP-CONA) detects quercetin as pri-miR-7/HuR interaction inhibitor that decreases α-synuclein levels
Source: Nucleic Acids Res. 2021 Jun 9;49(11):6456–73. doi: 10.1093/nar/gkab484 (PMC8216281; doi:10.1093/nar/gkab484)
Supplement: gkab484_Supplemental_File [file gkab484_supplemental_file.pdf]

SUPPLEMENTARY MATERIAL

Supplementary Figure 1

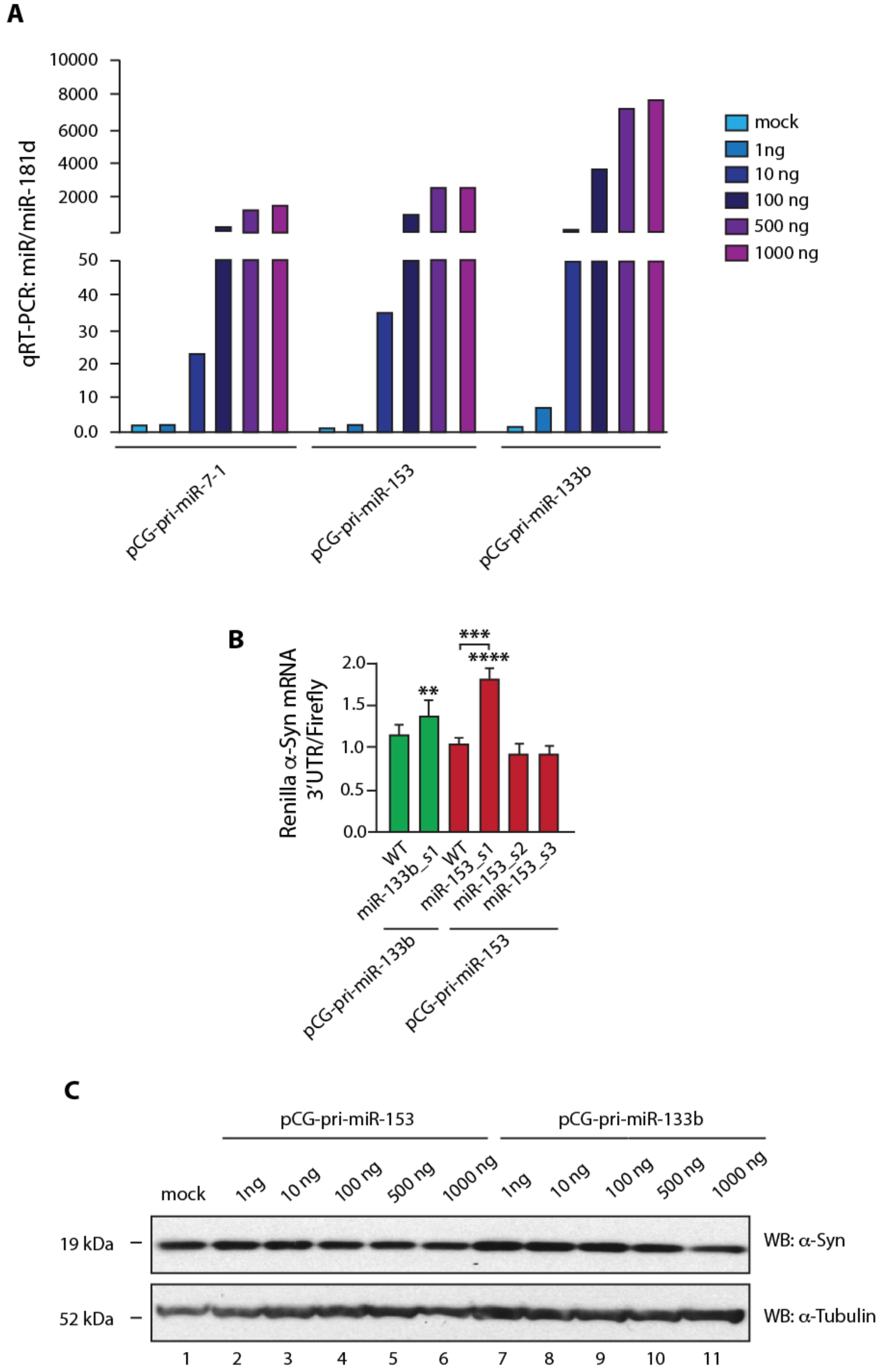

Supplementary Figure 1. MiR-7 is a major suppressor of α-Syn expression. (**A**) Upregulation of miRNAs after overexpression. Mature miR-7, miR-153 and miR-133b levels from HeLa cells transfected with increasing amount of corresponding pCG plasmids were determined by qRT-PCR and normalised to mock (no DNA transfected). (**B**) Single-nucleotide mutation of miR-133b and miR-153 binding site upregulated luciferase levels. The nucleotide on the 3rd position of miR-153 and miR-133b targeted seed regions on α-Syn mRNA 3'-UTR gene was mutated individually. The mutants were numbered according to the binding sites from 5' to 3' of α-Syn mRNA 3'-UTR. Luciferase levels were measured 48 hours after the co-transfection of pCG-pri-miR-133b or pCG-pri-miR-153-1 plasmids with reporters bearing wildtype or mutated α-Syn mRNA 3'-UTR gene. The luciferase levels were relative to co-transfection of pCG-pri-miR-9 with wildtype reporter (mock). Mean Renilla/firefly values and SEM from three independent repeats are shown. Statistically significant differences compared to mock were interpreted by SPSS one-way ANOVA, with post hoc LSD test, **P<0.01, ***P<0.001, ****P<0.0001. (**C**) MiR-153 and miR-133b exert mild inhibition on α-Syn expression.1: Mock HeLa cells without DNA transfected. 2-6: Increased amount of pCG-pri-miR-153-1 wase transfected into HeLa cells. 7-11: Increased amount of pCG-pri-miR-133b was transfected into HeLa cells. The expression of α-Syn and α-tubulin were detected by western blot 48 hours after transfection.

Supplementary Figure 2

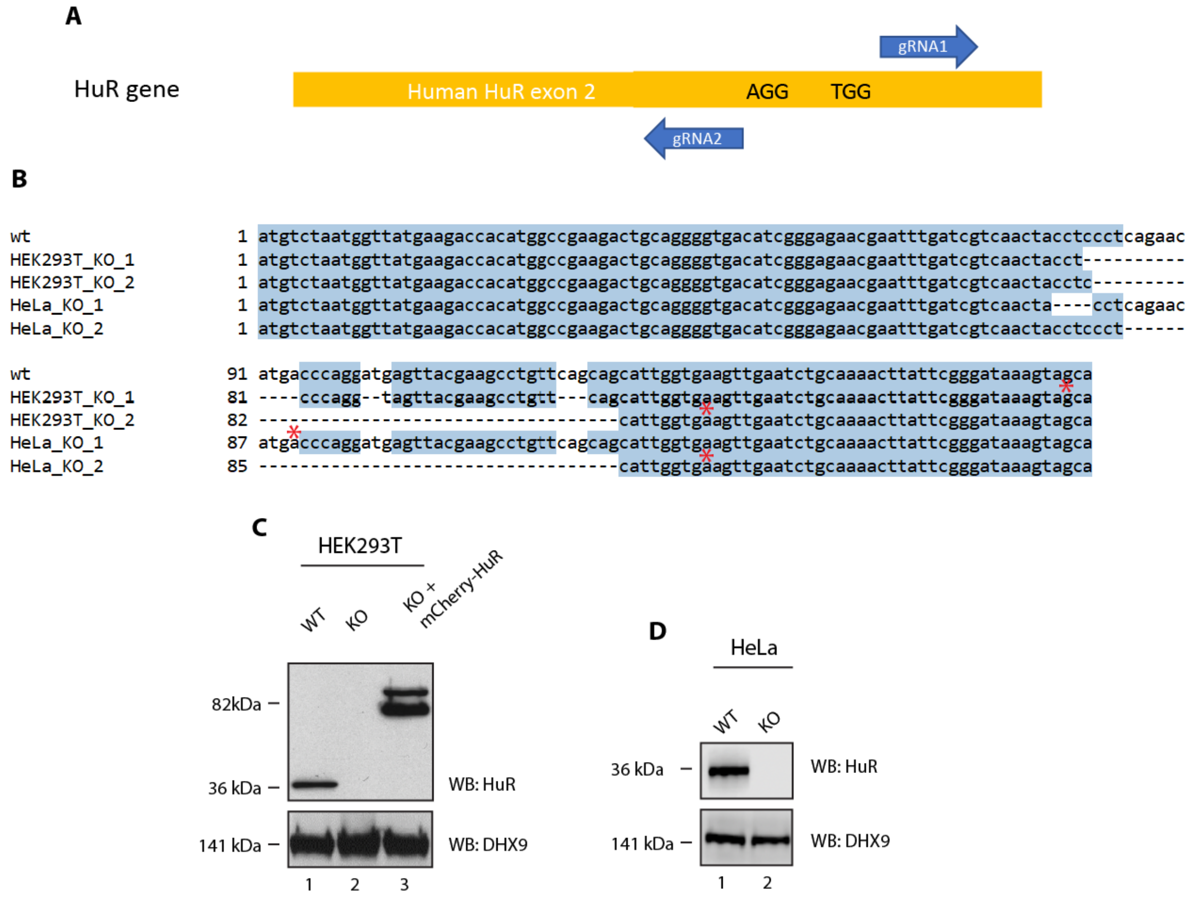

Supplementary Figure 2. HuR knockout and overexpression. (**A**) Design of HuR knockout by CRISPR-Cas9. Human HuR exon2 is shown in orange. A pair of guide RNAs are shown in blue arrows. PAM sequences are presented. (**B**) Sequence alignment of HuR knockouts. The DNA sequences of HuR exon2 of HEK293T and HeLa knockouts were aligned to the wildtype sequence in Clone Manager 10. Termination signals resulting from frame shifts are annotated by red stars. (**C**) Validation of HuR knockout and mCherry-HuR overexpression in HEK293T cells. The endogenous HuR and overexpressed mCherry-HuR levels were tested against HuR antibody in western blot. DHX9 was tested as a reference protein. 1: Wildtype HEK293T. 2: HuR KO HEK293T. 3: HuR KO HEK293T transfected with pJW99-HuR. (**D**) Validation of HuR knockout in HeLa cells. The HuR levels were tested against HuR antibody in western blot. DHX9 was tested as a reference protein. 1: Wildtype HeLa. 2: HuR KO HeLa.

Supplementary Figure 3

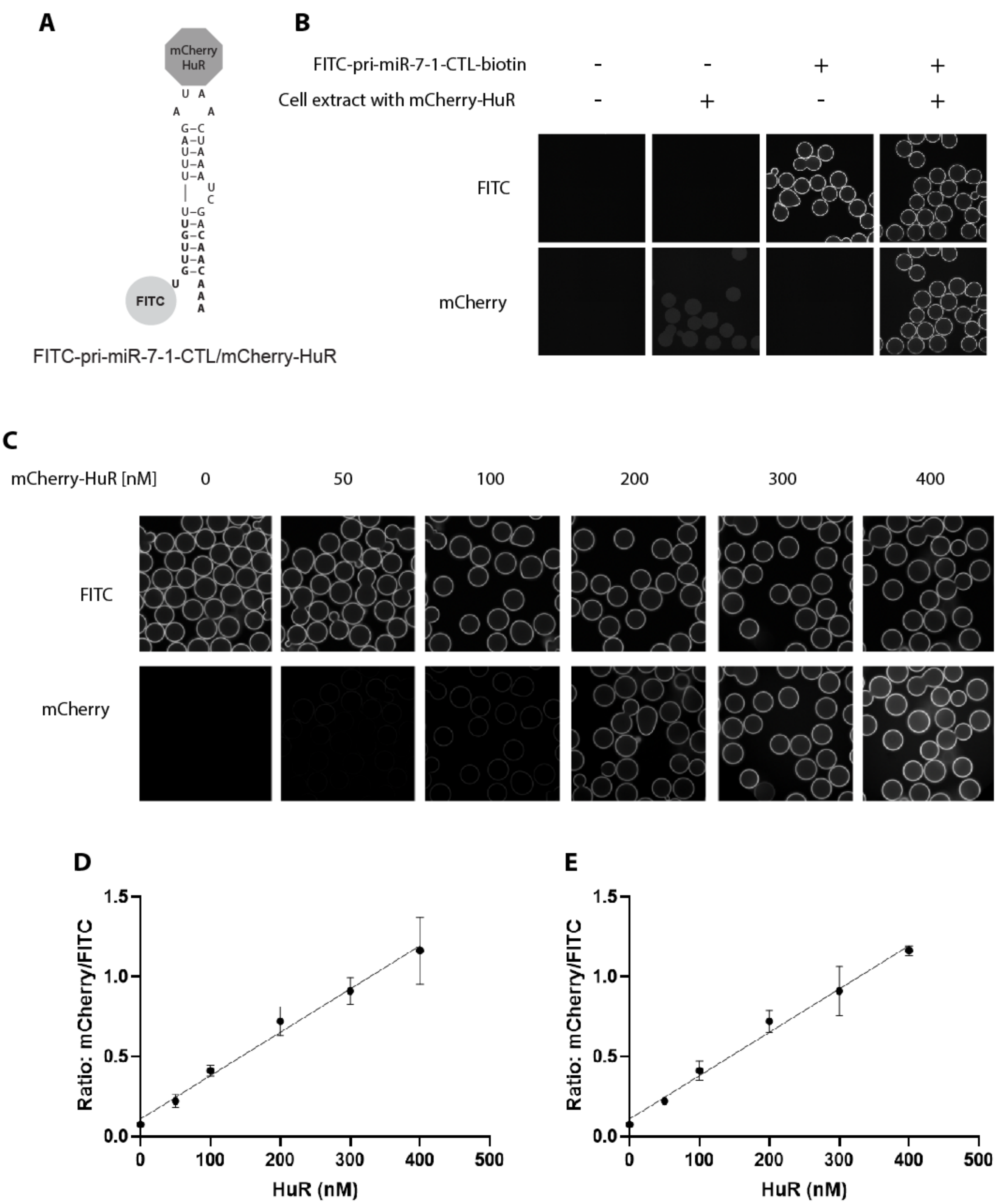

Supplementary Figure 3. Basic responses of RP-CONA in ImageXpress. (**A**) A diagram illustrating the interaction between FITC-pri-miR-7-1-CTL and mCherry-HuR. (**B**) Beads images of blank beads; blank beads incubated with cell lysates containing mCherry-HuR; FITC-pri-miR-7-1-CTL-

beads incubated in lysates-free buffer; and FITC-pri-miR-7-1-CTL-beads after mCherry-HuR pulldown. (**C- E**) RP-CONA senses the increase of mCherry-HuR. FITC-pri-miR-7-CTL-beads were incubated with cell lysates containing an increased concentration of mCherry-HuR. All the quantifications were obtained from three technical repeats. Mean mCherry/FITC ring intensities and maximum SD between the beads in each well (**D**), or SD between triplicates (**E**) are shown.

Supplementary Figure 4

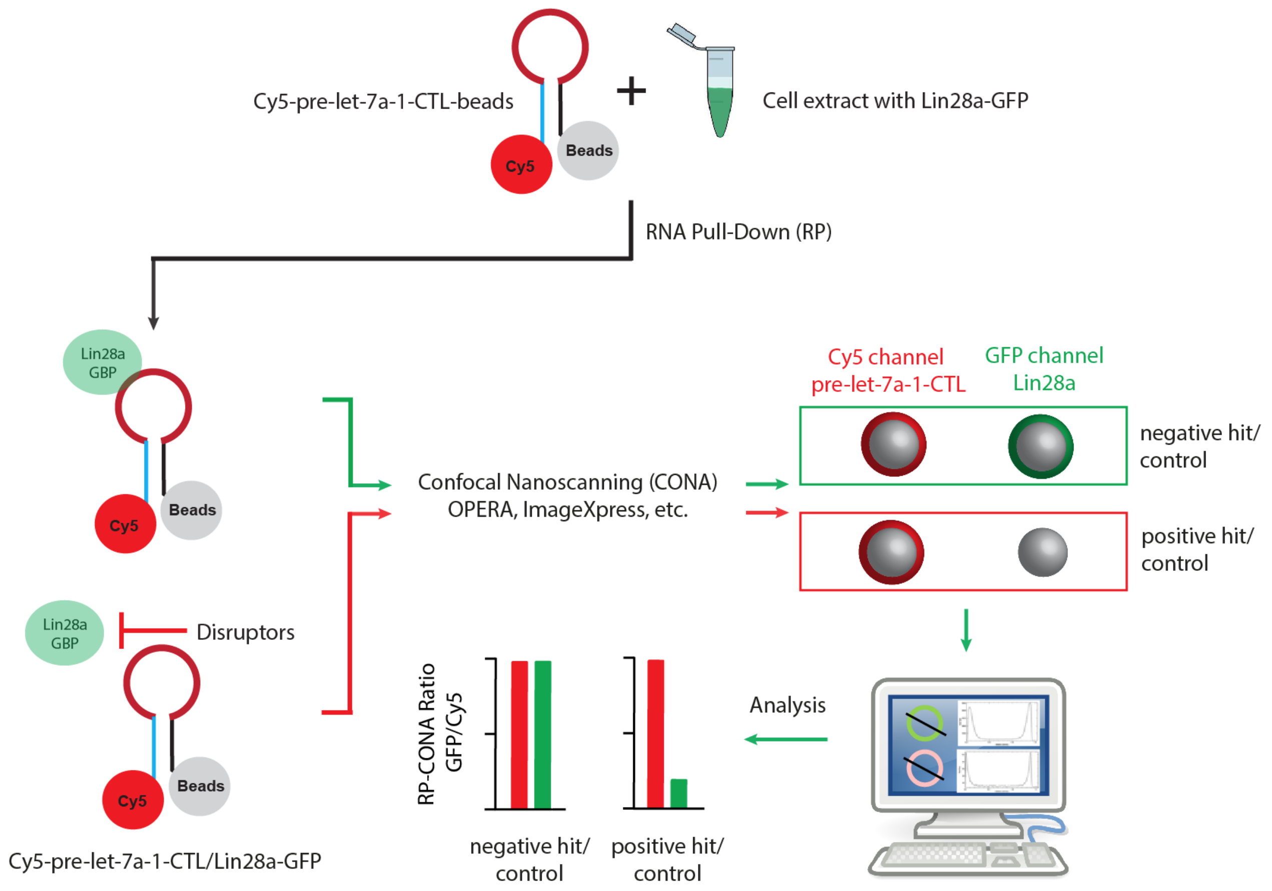

Supplementary Figure 4. The working scheme of RP-CONA targeting let-7/Lin28a. RNA Pull-down (RP): 5'-Cy5-pre-let-7a-1-CTL-biotin-3' is coupled to streptavidin coated agarose beads. Cell lysates are extracted from wildtype HEK293T cell overexpressing Lin28a-GFP and treated with small molecules. The RNA-coupled beads are incubated with cell lysates to pull-down Lin28a-GFP. Confocal Nanoscanning (CONA): Beads are imaged using a confocal image scanning platform. On-bead Cy5-pre-let-7a-1-CTL and Lin28a-GFP are detected with high sensitivity as fluorescent rings/halos on the outer shell of bead in corresponding detection channels. Inhibitors are able to attenuate the GFP fluorescence without affecting Cy5 signals. Analysis: The fluorescent rings are detected in imageJ. Three measurements are taken across each ring and generate the fluorescent intensity profiles to obtain arbitrary intensity values.

Supplementary Figure 5

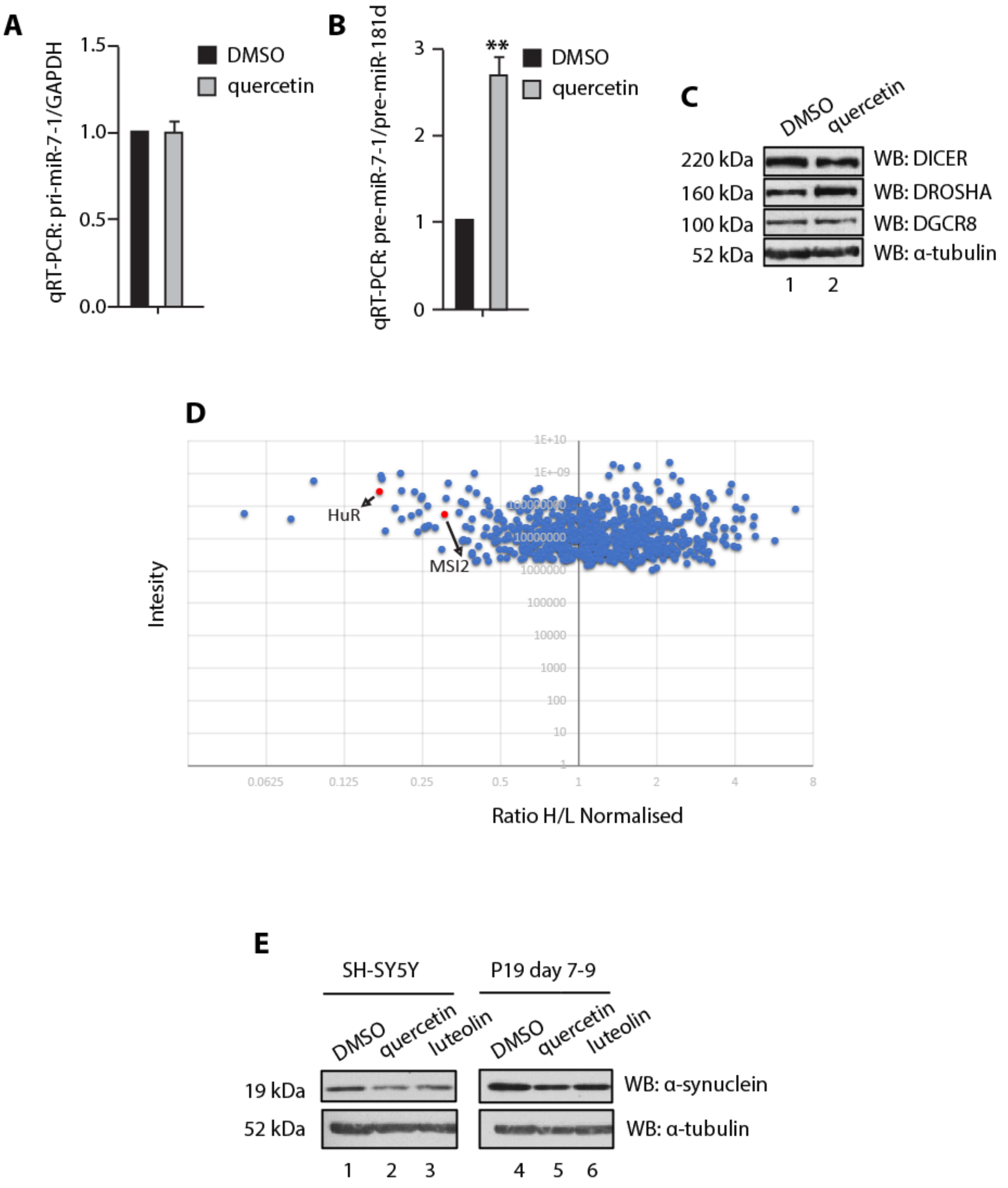

Supplementary Figure 5. Quercetin induces miR-7 biogenesis while inhibiting α-Syn expression. (**A, B**). Quercetin induces pri-miR-7 processing into pre-miR-7. Wildtype HeLa cells were treated with DMSO or 20 μM of quercetin and harvested 48 hours after treatment. Pri-miR-7-1 (**A**) and pre-miR-7-1 levels (**B**) were determined by qRT-PCR. Mean pri-miR-7-1/GAPDH, pre-miR-7-1/pre-

miR-181d and SEM from three independent repeats are shown. Statistically significant differences compared to DMSO were interpreted by SPSS independent sample t-test, ** $P<0.01$. (**C**). The effects of quercetin on the machinery of miRNA biogenesis. Wildtype HeLa cells were treated with 1: DMSO, 2: 20 μM of quercetin and harvested 48 hours after treatment. Levels of DICER, DROSHA, DGCR8 and α-tubulin were detected by western blot. (**D**). Quercetin inhibits HuR and MSI2 binding to pri-miR-7. RNA pull-down-SILAC mass spectrometry was performed using heavy HeLa extracts (^{13}C-arginine and ^{2}D-lysine) treated with 100 μM quercetin, and light HeLa extracts (no labelling) treated with DMSO. The experiment was repeated twice. Identified peptides with intensities larger than 1×10^6 are shown in blue dots. The corresponding normalised relative heavy/light intensity ratios are shown. Proteins with H/L ratios less than 0.5 were considered as enriched in light pull-down components. HuR and MSI2 are highlighted with red dots. (**E**). Quercetin downregulates α-Syn expression in neuron-like cells. SH-SY5Y cells were treated with 1: DMSO, 2: 20 μM of quercetin or 3: luteolin and harvested 48 hours after treatment. P19 cells were differentiated by retinoic acid cells. On the Day 17 of differentiation, the cells were treated with 4: DMSO, 5: 20 μM of quercetin or 6: luteolin and harvested 48 hours after treatment. Levels of α-Syn and α-tubulin were detected by western blot. The experiments were repeated for three times.

Supplementary Figure 6

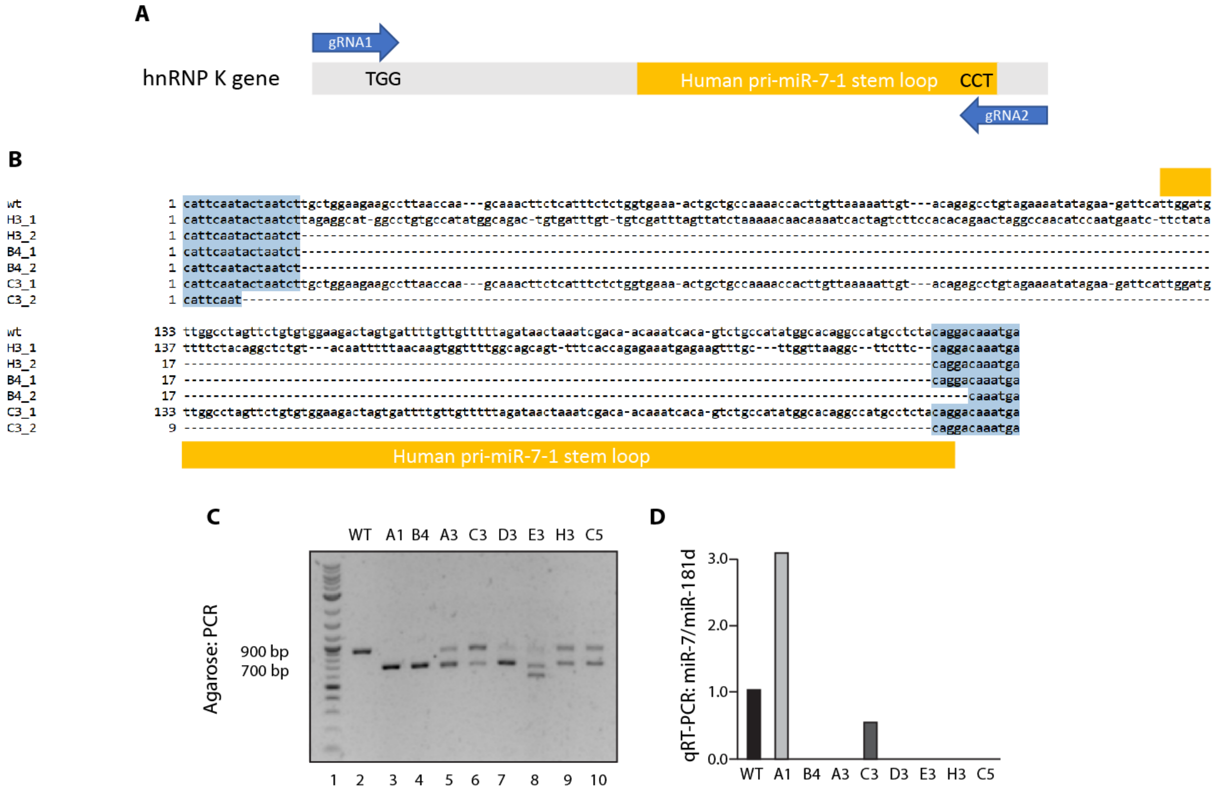

Supplementary Figure 6. MiR-7-1 knockout by CRISPR-Cas9. (**A**) Design of miR-7-1 knockout by CRISPR-Cas9. Human pri-miR-7-1 stem loop sequence is shown in orange. Other regions are in grey. A pair of guide RNAs flanking the pri-miR-7-1 stem loop gene are shown in blue arrows. PAM sequences are presented. (**B**) Sequence alignment of miR-7-1 knockouts. Sequencing results of miR-7-1 knockouts were aligned to the same region of wildtype HeLa gene in Clone Manager 10. Regions originally encoding pri-miR-7-1 stem loop are indicated. B4 is a miR-7-1$^{-/-}$ as the miR-7-1 stem loop was deleted in both alleles. C3 is a miR-7-1$^{+/-}$ as miR-7-1 was deleted in one allele while the other allele is intact. H3 has miR-7-1 deleted in one allele. The other allele, however, the miR-7-1 stem loop was re-assembled in an opposite orientation. Therefore, H3 is a miR-7-1$^{-/-}$. (**C**) Genotyping and characterisation of miR-7-1 KO HeLa. Genomic DNA was PCR amplified using a pair of primers flanking pri-miR-7-1 stem loop. The deleted stem loop region was expected to be around 200 bp. Bands around 700 bp represent potential KO. (**D**) MiR-7 level of miR-7-1 KO HeLa. Mature miR-7 and miR-181d levels of wildtype HeLa or miR-7-1 knockouts were determined by qRT-PCR.

Supplementary Figure 7

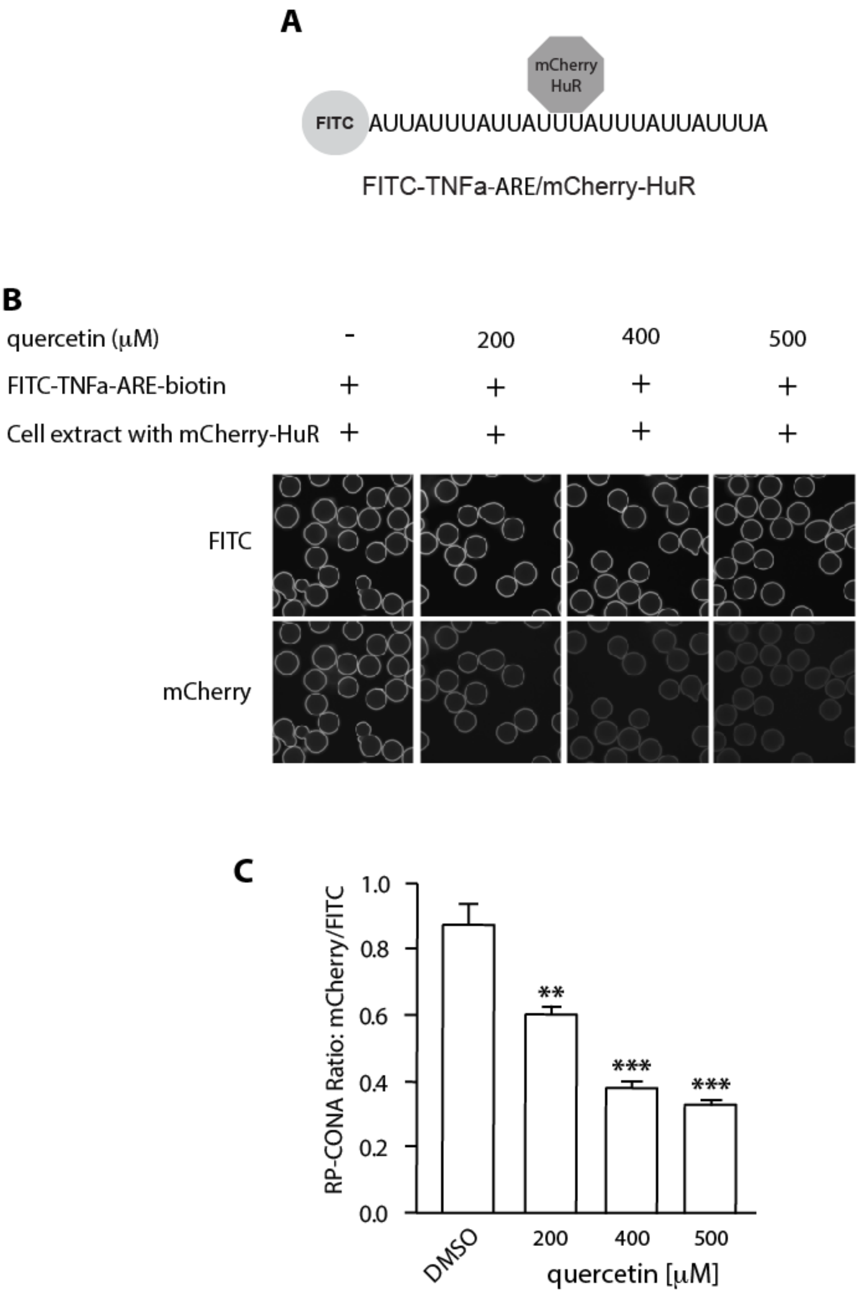

Supplementary Figure 7. Quercetin inhibits pre-let-7a-1/Lin28a interaction in RP-CONA (**A**). A diagram illustrating the interaction between Cy5-pre-let7a-1-CTL and Lin28a-GFP. (**B, C**). Cell lysates containing 300 nM of GFP-Lin28a were treated with 100 μM of quercetin, prior to pull-down by Cy5-pre-let7a-1-CTL-beads. Mean GFP/Cy5 ring intensities and SD between triplicates are shown. Statistically significant differences compared to HuR antibody treated samples were interpreted by SPSS independent sample t-test, ** P<0.01.

Supplementary Figure 8

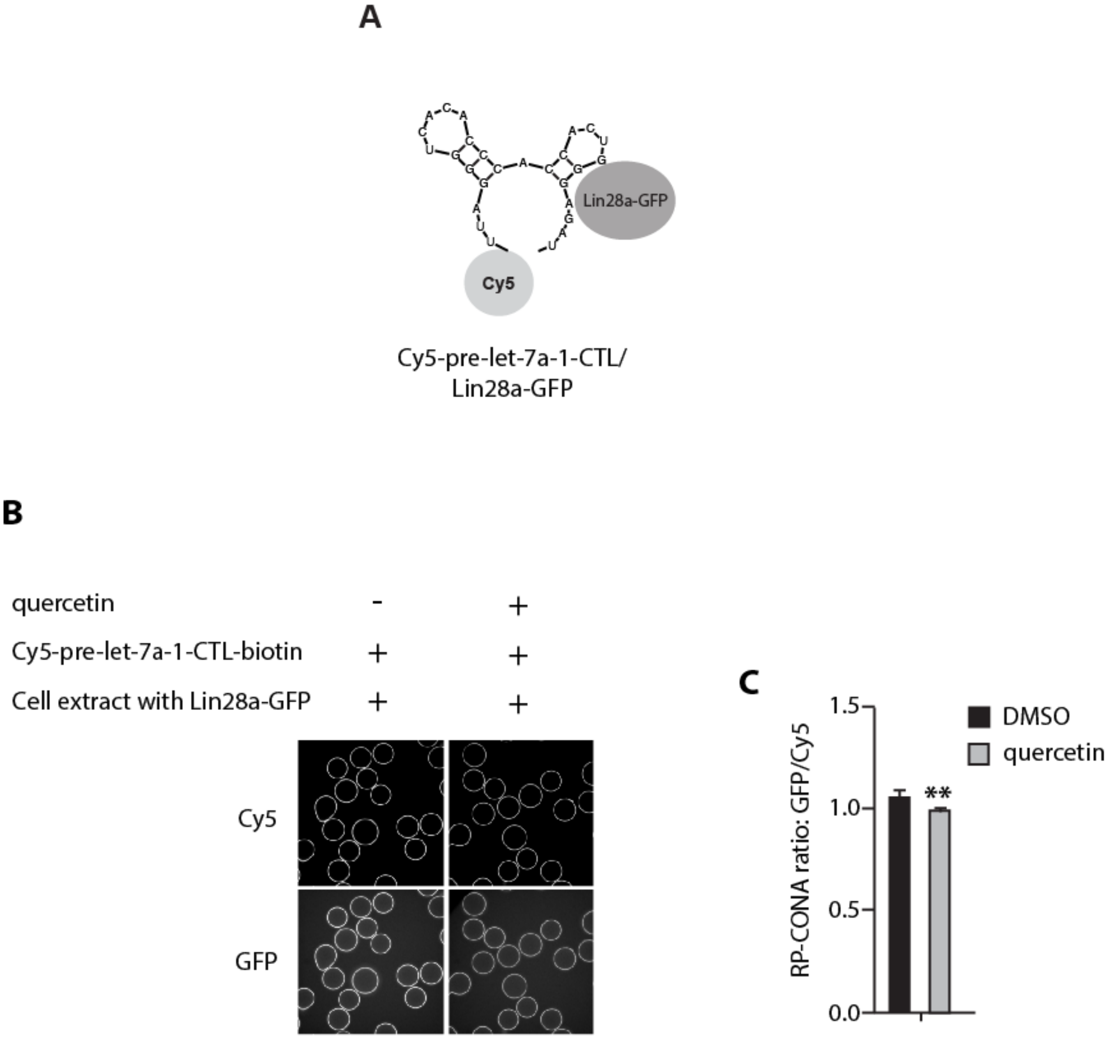

Supplementary Figure 8. Quercetin inhibits TNFα-ARE/HuR in RP-CONA. (**A**). A diagram illustrates the interaction between FITC-TNFα-ARE with mCherry-HuR. (**B, C**). Cell extracts containing 50 nM of mCherry-HuR were pre-incubated with an increased concentration of quercetin, prior pull-down by FITC-TNFα-ARE-beads. Mean mCherry/FITC ring intensities and SD between triplicates are shown. Statistically significant differences compared to DMSO were interpreted by SPSS independent sample t-test, ** $P<0.01$, ***$P<0.001$.

METHODS

Quantification of pri and pre-miRs

Total RNA was treated by TURBO™ DNase (Thermo) to remove contaminant DNA. Pri-miRs were quantified by qRT-PCR with primers upstream the stem-loop structures, using the GoTaq® 1-Step RT-qPCR System (Promega). To test pre-miRs, total RNA was fractionated using a 6% Urea-PAGE gel. RNAs between the bromophenol blue (26 nt) and xylene cyanol (106 nt) dye were purified to remove pri and mature miRs. The purified RNAs were reversed transcribed following the instructions of the miScript II RT Kit for pre-miR. Then pre-miRs were quantified following the instructions of the SYBR Green PCR Kit (QIAGEN) using mature miR primers and the universal reverse primer provided.

RNA pull-down-SILAC mass spectrometry (RP-SMS)

RP-SMS was performed according to our previous published method (1). Heavy HeLa extracts (^{13}C-labelled arginine and ^{2}D-labelled lysine) were treated with 100 µM quercetin, and light HeLa extracts (no labelling) were treated with equal volume of DMSO. Both cell extracts were incubated with pri-miR-7-1-CTL-linked beads, respectively. The pull-down components from heavy and light extracts were combined during the last beads washing and loaded onto an SDS-PAGE gel. The mass spectrometry analysis was performed as described previously (1).

qRT-PCR primers

pri-miR-7-1-F	GCCATGGTGTCTCAACCTTT
pri-miR-7-1-R	GTGGTTTTGGCAGCAGTTTT
GAPDH-F	AATCCCATCACCATCTTCCA
GAPDH-R	TGGACTCCACGACGTACTCA
miR-7-5p	TGGAAGACTAGTGATTTTGTTGTT
miR-153-3p	TTGCATAGTCACAAAAGTGATC
miR-133b-3p	TTTGGTCCCCTTCAACCAGCTA
miR-9-5p	TCTTTGGTTATCTAGCTGTATGA
miR-181d-5p	AACATTCATTGTTGTCGGTGGGT
18S-F	GTAACCCGTTGAACCCCATT
18S-R	CCATCCAATCGGTAGTAGCG

Cloning primers

miR-133b_s1-F	GAAACACTTAAACAAAAAGTTCTTTA
miR-133b_s1-R	GTCCCAAATAAACTATTAAGATATAT
miR-153_s1-F	CCACCTATAAATACTAAATATGAAATT
miR-153_s1-R	ATAGTTTCATGCTCACATATTTTTAA

miR-153_s2-F	CCACTGGTTCCTTAAGTGGCTG
miR-153_s2-R	ATACCAAAACACACTTCTGGCA
miR-153_s3-F	CCACTAGTGTGAGATGCAAACA
miR-153_s3-R	AGAGATTCTGAAAAAGACCCCA
Pre-let-7a-1_T7-F	TAATACGACTCACTATAGGTGAGGTAGTAGGTTGTATAG
Pre-let-7a-1_T7-R	GAAAGACAGTAGATTGTATA

1. Choudhury, N.R. and Michlewski, G. (2018) Quantitative identification of proteins that influence miRNA biogenesis by RNA pull-down-SILAC mass spectrometry (RP-SMS). *Methods*.
